# TLR7/8 stress response drives histiocytosis in SLC29A3 disorders

**DOI:** 10.1084/jem.20230054

**Published:** 2023-07-18

**Authors:** Takuma Shibata, Ryota Sato, Masato Taoka, Shin-Ichiroh Saitoh, Mayumi Komine, Kiyoshi Yamaguchi, Susumu Goyama, Yuji Motoi, Jiro Kitaura, Kumi Izawa, Yoshio Yamauchi, Yumiko Tsukamoto, Takeshi Ichinohe, Etsuko Fujita, Ryosuke Hiranuma, Ryutaro Fukui, Yoichi Furukawa, Toshio Kitamura, Toshiyuki Takai, Arinobu Tojo, Mamitaro Ohtsuki, Umeharu Ohto, Toshiyuki Shimizu, Manabu Ozawa, Nobuaki Yoshida, Toshiaki Isobe, Eicke Latz, Kojiro Mukai, Tomohiko Taguchi, Hiroaki Hemmi, Shizuo Akira, Kensuke Miyake

**Affiliations:** 1 Division of Innate Immunity, Department of Microbiology and Immunology, The Institute of Medical Science, The University of Tokyo, Tokyo, Japan; 2 Department of Chemistry, Graduate School of Science, Tokyo Metropolitan University, Tokyo, Japan; 3 Department of Dermatology, Jichi Medical University, Tochigi, Japan; 4 Division of Clinical Genome Research, The Institute of Medical Science, The University of Tokyo, Tokyo, Japan; 5 Division of Molecular Oncology, Department of Computational Biology and Medical Sciences, Graduate School of Frontier Sciences, The University of Tokyo, Tokyo, Japan; 6 Atopy Research Center, Graduate School of Medicine, Juntendo University, Tokyo, Japan; 7 Department of Mycobacteriology, Leprosy Research Center, National Institute of Infectious Diseases, Tokyo, Japan; 8 Division of Viral Infection, Department of Infectious Disease Control, International Research Center for Infectious Diseases, The Institute of Medical Science, The University of Tokyo, Tokyo, Japan; 9 Division of Cellular Therapy, The Institute of Medical Science, The University of Tokyo, Tokyo, Japan; 10 Department of Experimental Immunology, Institute of Development, Aging and Cancer, Tohoku University, Sendai, Japan; 11 Department of Hematology and Oncology, Research Hospital, The Institute of Medical Science, The University of Tokyo, Tokyo, Japan; 12 Graduate School of Pharmaceutical Sciences, The University of Tokyo, Tokyo, Japan; 13 Laboratory of Developmental Genetics, Center for Experimental Medicine and Systems Biology, The Institute of Medical Science, The University of Tokyo, Tokyo, Japan; 14 Institute of Innate Immunity, University Hospital Bonn, University of Bonn, Bonn, Germany; 15 Department of Integrative Life Sciences, Laboratory of Organelle Pathophysiology, Graduate School of Life Sciences, Tohoku University, Sendai, Japan; 16 Laboratory of Immunology, Faculty of Veterinary Medicine, Okayama University of Science, Imabari, Japan; 17 Laboratory of Host Defense, World Premier Institute—Immunology Frontier Research Center (WPI-IFReC), Osaka University, Osaka, Japan; 18 Department of Host Defense, Research Institute for Microbial Diseases (RIMD), Osaka University, Osaka, Japan

## Abstract

Loss-of-function mutations in the lysosomal nucleoside transporter SLC29A3 cause lysosomal nucleoside storage and histiocytosis: phagocyte accumulation in multiple organs. However, little is known about the mechanism by which lysosomal nucleoside storage drives histiocytosis. Herein, histiocytosis in *Slc29a3*^−/−^ mice was shown to depend on Toll-like receptor 7 (TLR7), which senses a combination of nucleosides and oligoribonucleotides (ORNs). TLR7 increased phagocyte numbers by driving the proliferation of Ly6C^hi^ immature monocytes and their maturation into Ly6C^low^ phagocytes in *Slc29a3*^−/−^ mice. Downstream of TLR7, FcRγ and DAP10 were required for monocyte proliferation. Histiocytosis is accompanied by inflammation in SLC29A3 disorders. However, TLR7 in nucleoside-laden splenic monocytes failed to activate inflammatory responses. Enhanced production of proinflammatory cytokines was observed only after stimulation with ssRNAs, which would increase lysosomal ORNs. Patient-derived monocytes harboring the G208R *SLC29A3* mutation showed enhanced survival and proliferation in a TLR8-antagonist-sensitive manner. These results demonstrated that TLR7/8 responses to lysosomal nucleoside stress drive SLC29A3 disorders.

## Teaser

Loss-of-function mutations in the gene encoding the lysosomal nucleoside transporter SLC29A3 cause histiocytic diseases, collectively termed SLC29A3 disorders. Shibata et al. show that histiocytosis is driven by mouse TLR7 and human TLR8, endosomal sensors for nucleosides and oligoribonucleotides, in mice and humans, respectively.

## Introduction

Toll-like receptor 7 (TLR7) and TLR8 are endosomal ssRNA sensors that initiate innate immune responses during viral and bacterial infections (Diebold et al., 2004; Heil et al., 2004). Loss-of-function mutations in *TLR7* are a genetic risk factor for the development of COVID-19 pneumonia (Brown et al., 2022), whereas gain-of-function mutations in *TLR7* and *TLR8* drive immune disorders such as systemic lupus erythematosus (Aluri et al., 2021; Brown et al., 2022). Their structures indicate that they respond to RNA degradation products; TLR7 binds to guanosine (Guo) or 2′-deoxyguanosine (dGuo), as well as to uridine (Urd)-containing oligoribonucleotides (ORNs), whereas TLR8 interacts with Urd- and purine-containing ORNs (Shibata et al., 2016; Tanji et al., 2015; Zhang et al., 2016). These structures demonstrate that ssRNA recognition by TLR7 and TLR8 depends on RNA degradation. For example, RNA degradation by the endosomal RNase, RNase T2, is required for TLR7 and TLR8 responses (Greulich et al., 2019; Liu et al., 2021; Ostendorf et al., 2020).

RNA degradation in endosomes/lysosomes proceeds up to nucleosides, which are then transported to the cytoplasm for further degradation. Nucleosides are transported across the membrane through the SLC28 and SLC29 transporter families (Baldwin et al., 2004). While SLC28 family members mediate active transport in epithelial tissues such as the small intestine and kidney, SLC29 family members enable passive transport in broader tissues. SLC29A3, also known as ENT3, is a lysosomal nucleoside transporter that is abundantly expressed in macrophages (Baldwin et al., 2005). Loss-of-function mutations in SLC29A3 cause monogenic diseases, including H syndrome, Faisalabad histiocytosis, pigmented hypertrichosis with insulin-dependent diabetes mellitus syndrome, and familial Rosai-Dorfman disease (Cliffe et al., 2009; Molho-Pessach et al., 2008; Morgan et al., 2010). These SLC29A3 disorders are characterized by histiocytosis: mononuclear phagocyte accumulation in multiple organs. Histiocytosis also develops in *Slc29a3*^*−/−*^ mice, where adenosine (Ado) accumulates in the lysosomes of macrophages because of impaired nucleoside export to the cytoplasm (Hsu et al., 2012). However, the mechanisms by which lysosomal Ado storage increases the number of phagocytes have not yet been elucidated.

We hypothesized that Guo, dGuo, and Urd accumulate in the compartments where TLR7 and TLR8 are localized and activate TLR7 and TLR8 to drive histiocytosis in SLC29A3 disorders. We observed an accumulation of Guo and dGuo in *Slc29a3*^*−/−*^ monocytes/macrophages. Immature Ly6C^hi^ monocytes TLR7-dependently proliferate and mature into Ly6C^low^ phagocytes. In SLC29A3 disorders, histiocytosis accompanies inflammation (Molho-Pessach et al., 2014; Rafiq et al., 2017). SLC29A3 deficiency did not induce constitutive production of proinflammatory cytokines in macrophages and required ORN-generating ssRNA to drive proinflammatory cytokine production in macrophages. Patient-derived monocytes harboring G208R *SLC29A3* mutation showed higher survival and proliferation in the presence of M-CSF and produced larger amounts of IL-6 upon ssRNA stimulation than those derived from healthy subjects. G208R *SLC29A3* monocytes expressed TLR8, and a TLR8 antagonist inhibited the survival/proliferation of patient-derived macrophages. Moreover, human TLR8 expressed in *Slc29a3*^−/−^ *Tlr7*^−/−^ mice caused histiocytosis. These results demonstrated that TLR7 and TLR8 responses to nucleosides drive SLC29A3 disorders.

## Results

### TLR7-dependent histiocytosis in *Slc29a3*^−/−^ mice

**Figure S1.**
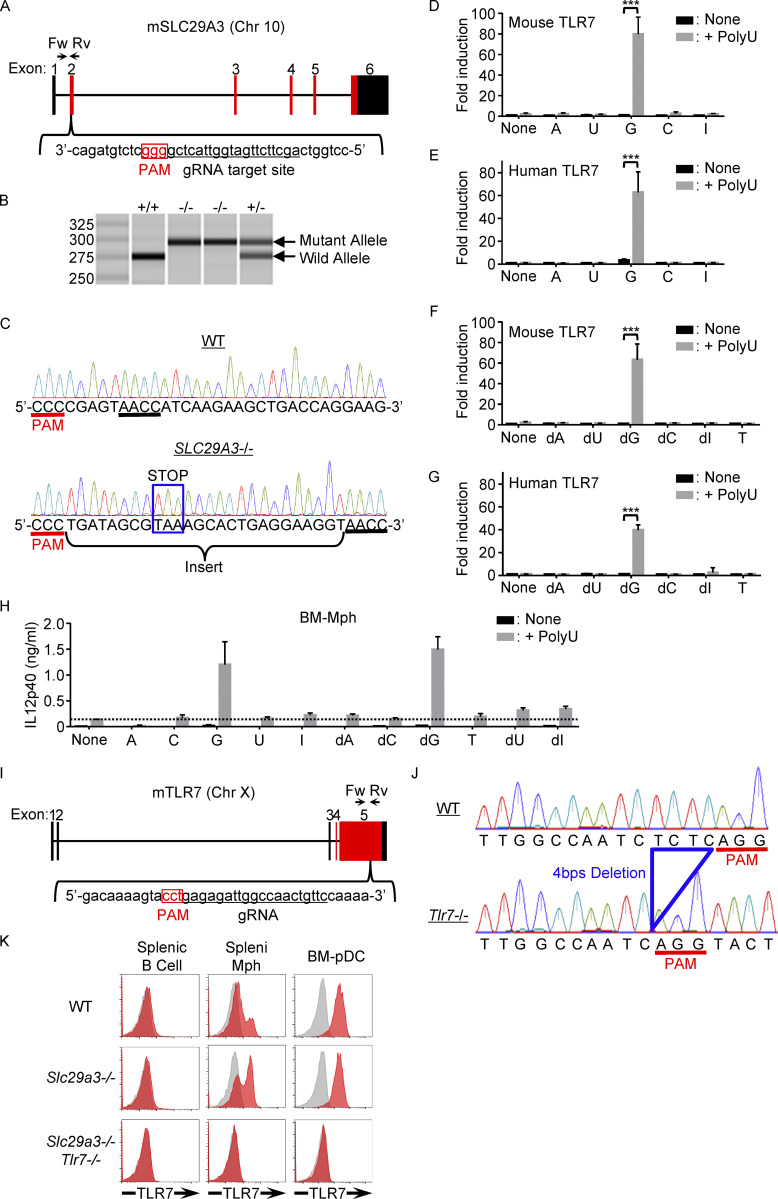
**Generation of *Slc29a3***^**−/−**^ **and *Tlr7***^**−/−**^ **mice. (A and I)** Genomic configuration of *Slc29a3* and *Tlr7* genes showing 20mer gRNA target sites to introduce a mutation into exon 2 of *Slc29a3* and exon 5 of *Tlr7*. The PAM sequence is highlighted by the red box. **(B)** Genomic PCR with the primer set (Fw and Rv) shown in A reveals an insertional mutation in the targeted allele of *Slc29a3*. **(C)** Direct sequencing of the gRNA target site of *Slc29a3*. The inserted sequence containing the stop codon is shown by the blue box. **(D–G)** NF-κB reporter assay using HEK293T cells transfected with TLR7 and human Unc93B1. Transfected cells were left unstimulated or stimulated with indicated nucleoside ligands (100 μM) with or without polyU (5 μg/ml). The results are represented as mean values ± SD of triplicates. **(H)** IL-12 p40 production by BM-Mphs left unstimulated or stimulated with a combination of polyU (1 μg/ml) and the indicated nucleoside (100 μM). The results are represented by mean values ± SD of triplicates. **(J)** Sequence data of the gRNA target site on the *Tlr7* allele showing a 4-bp deletion in the fifth exon of *Tlr7* (blue). **(K)** FACS analyses show the lack of TLR7 protein in splenic B cells, splenic monocytes, and BM-derived pDCs in *Slc29a3*^‒/‒^ *Tlr7*^‒/‒^ mice. Red and gray histograms represent intracellular staining with and without anti-TLR7 mAb, respectively. The data shown in D–H and K are representative of at least three independent experiments. *P < 0.05, **P < 0.01, and ***P < 0.001.

**Figure 1.**
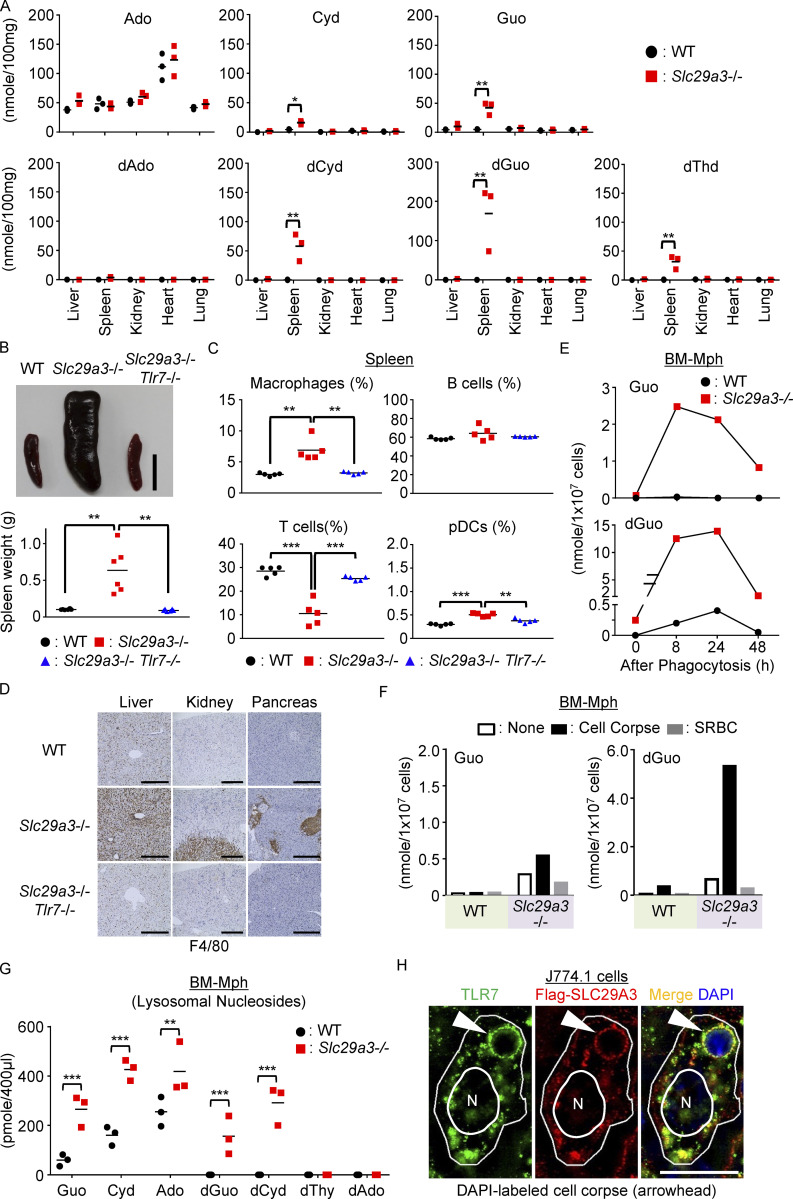
**TLR7-dependent histiocytosis in *Slc29a3***^**‒/‒**^ **mice. (A)** Amounts of nucleosides in the indicated organs. Each dot represents a value (nanomole/100 mg tissue) from each mouse (*n* = 3). **(B)** Representative spleen images from 6-mo-old mice (top). Scale bar, 1 cm. The bottom panel shows the spleen weight (*n* = 6). **(C)** Percentages of NK1.1^‒^ Ly6G^‒^ CD11b^+^ monocytes, CD19^+^ B cells, CD3ε^+^ T cells, and PDCA1^+^ pDCs in the CD45.2^+^ splenocytes from the indicated mice (*n* = 5). **(D)** Immunohistochemistry showing F4/80 expression in indicated organs of tested mice. Scale bar, 400 μm. **(E and F)** Amounts of accumulated nucleosides (nanomoles) in 10^7^ cells of WT and *Slc29a3*^−/−^ BM-Mphs after treatment with 10^8^ dying thymocytes (cell corpse) or 10^9^ SRBCs for the indicated hours (E) or 24 h (F) were evaluated by LC-MS. The experiments were performed twice and yielded the same results. **(G)** Amounts of nucleosides in lysosomal fractions from 10^8^ BM-Mphs (*n* = 3). **(H)** Staining of TLR7 and Flag-SLC29A3 in the J774.1 macrophage cell line at 1 h after phagocytosis of the DAPI-labeled cell corpse. Arrowheads indicate a phagosome containing cell corpses. Scale bar, 10 μm. The data shown in D and H are representative of at least four independent experiments. *P < 0.05, **P < 0.01, and ***P < 0.001.

**Figure S2.**
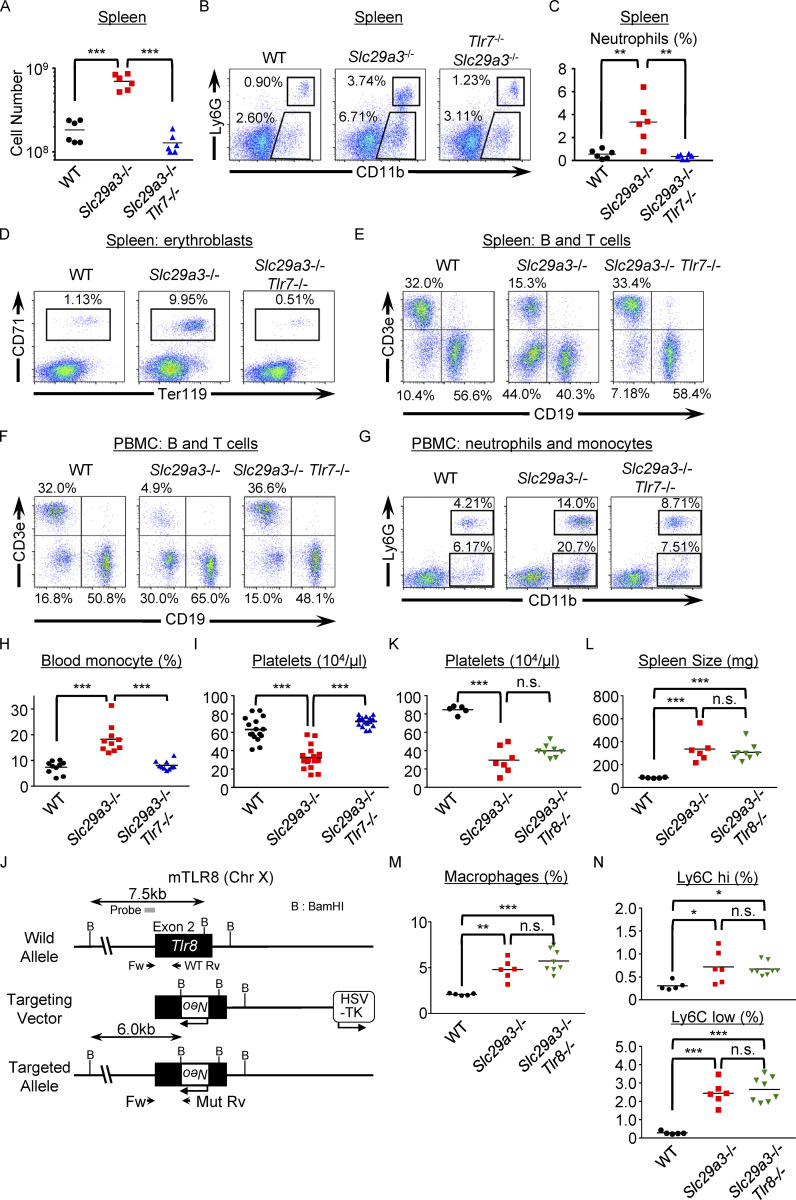
**TLR7-dependent monocytosis in *Slc29a3***^**−/−**^ **mice. (A)** Dot plots show the number of splenocytes in the indicated mice (*n* = 6). **(B)** Representative FACS analyses of Siglec F^−^ NK1.1^−^ splenocytes to show their expression of CD11b and Ly6G. **(C)** Each dot shows the percentage of neutrophils in whole splenocytes from the indicated mice (*n* = 6). **(D and E)** Representative FACS analyses of CD71^+^ Ter119^+^ erythroblasts (D) or CD19^+^ B and CD3ε^+^ T cells (E) in the spleen. **(F and G)** Representative FACS analyses of B and T cells (F) or neutrophils and monocytes (G) in PBMCs. **(H and I)** Percentages of CD11b^+^ Ly6G^‒^ monocytes (*n* = 10) in PBMCs (H) and platelet counts (*n* = 16) in the peripheral blood (I) of 3-mo-old mice. **(J)** Schematic representation of the *Tlr8* gene targeting strategy. The filled and open boxes represent the second exon of the *Tlr8* gene and the neomycin resistance (*Neo*) gene, respectively. The 6 and 7.5 kb DNA fragments detected by a probe (gray box) in Southern blot screening are also shown. B, BamH I. **(K and L)** Percentages of platelet counts in the peripheral blood (K) and spleen size (L) of 4-mo-old mice (*n* = 5–8). **(M and N)** The percentages of NK1.1^−^ Ly6G^−^ CD11b^+^ macrophages (M) and Ly6C^hi^ and Ly6C^low^ macrophages (N) in the CD45.2^+^ splenocytes from the indicated mice (*n* = 5–8). *P < 0.05, **P < 0.01, and ***P < 0.001.

*Slc29a3*^−/−^ mice were obtained (Fig. S1, A–C), and various organs of these mice were examined by liquid chromatography–mass spectrometry (LC-MS) to evaluate nucleoside accumulation. We found significant increases in cytidine (Cyd), Guo, 2′-deoxycytidine (dCyd), dGuo, and thymidine (dThd) in the spleens of lysosomal nucleoside transporter-deficient *Slc29a3*^‒/‒^ mice (Fig. 1 A). As TLR7 responds to Guo and dGuo but not to other nucleosides or deoxyribonucleosides (Fig. S1, D–H; Shibata et al., 2016), accumulation of Guo and dGuo in *Slc29a3*^−/−^ mice might activate TLR7. We generated *Slc29a3*^‒/‒^*Tlr7*^‒/‒^ mice to evaluate the role of TLR7 in histiocytosis (Fig. S1, I–K). Consistent with the previous report (Hsu et al., 2012), the spleens of *Slc29a3*^‒/‒^ mice were larger and heavier than those of WT mice due to increased cell number (Fig. 1 B and Fig. S2 A). Concerning cell type–specific changes in the spleen, the increase in numbers was restricted to macrophages, neutrophils, erythroblasts, and plasmacytoid dendritic cells (pDCs), but not T cells or B cells (Fig. 1 C and Fig. S2, B–H). Peripheral blood platelet counts decreased, probably, due to premature clearance by accumulated macrophages (Fig. S2 I). Macrophage accumulation was observed not only in the spleen but also in the liver, the medulla of the kidney, and the patchy areas of the pancreas (Fig. 1 D). All these changes were dependent on TLR7, as demonstrated in the *Slc29a3*^‒/‒^ *Tlr7*^‒/‒^ mice (Fig. 1, B–D; and Fig. S2).

Mouse TLR8 does not respond to nucleosides and ssRNAs (Heil et al., 2004), and negatively regulates TLR7 (Demaria et al., 2010), suggesting that mouse TLR8 might inhibit TLR7-dependent histiocytosis. To study the role of TLR8 in *Slc29a3*^*−/−*^ mice, we generated *Slc29a3*^*−/−*^ *Tlr8*^*−/−*^ mice, where the phenotypes of splenomegaly and thrombocytopenia were not altered (Fig. S2, J–N). Therefore, TLR8 did not impact histiocytosis in *Slc29a3*^*−/−*^ mice.

### Cell corpse phagocytosis increases nucleoside storage

**Figure S3.**
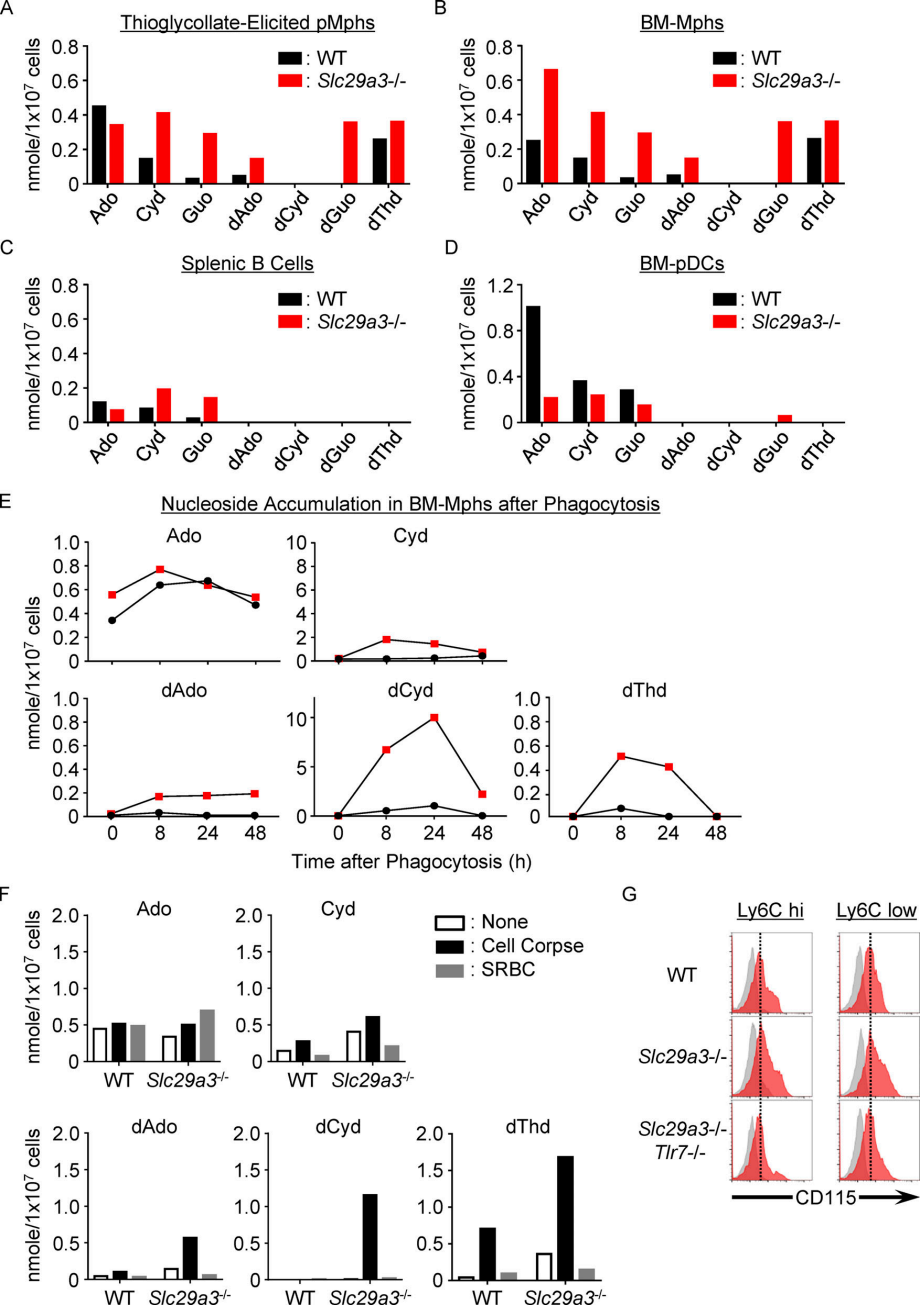
**Nucleoside storage in *Slc29a3***^**−/−**^ **phagocytes. (A–D)** Amount of nucleosides (in nanomole) in 10^7^ thioglycolate-elicited pMphs (A), BM-Mphs (B), splenic B cells (C), and BM-pDCs (D) from WT (black) and *Slc29a3*^−/−^ (red) mice. **(E)** Amount of nucleosides (in nanomoles) in 10^7^ BM-Mphs of WT and *Slc29a3*^−/−^ mice at the indicated time points after treatment with 10^8^ dying thymocytes (cell corpse). **(F)** Amount of nucleosides (nanomoles) in 10^7^ BM-Mphs of WT and *Slc29a3*^−/−^ mice after 1-d treatment with 10^8^ dying thymocytes or 10^9^ SRBC. **(G)** Red histograms show cell surface expression of CD115 in Ly6C^hi^ and Ly6C^low^ monocytes in the spleens of WT, *Slc29a3*^‒/‒^, and *Slc29a3*^−/−^ *Tlr7*^−/−^ mice. Gray histograms show staining with control mAb. The data shown in A–G are representative of at least two independent experiments.

Nucleoside storage was also observed in professional phagocytes, such as thioglycolate-elicited peritoneal macrophages (pMphs) and bone marrow (BM)–derived macrophages (BM-Mphs; Fig. S3, A and B). In contrast, we observed much smaller nucleoside increases in other TLR7-expressing immune cells such as splenic B cells and BM-pDCs (Fig. S3, C and D). Given that B cells and BM-pDCs are less phagocytic (Aderem and Underhill, 1999; Dalgaard et al., 2005), phagocytosis might increase nucleoside storage. To address this possibility, we exposed dying thymocytes (cell corpses) to BM-Mphs (Fig. 1 E and Fig. S3 E). We observed increases in nucleosides such as dGuo and dCyd, which peaked at 8–24 h after cell corpse treatment. At 48 h, the levels of dGuo returned to normal in WT BM-Mphs but remained high in *Slc29a3*^−/−^ BM-Mphs. A lower, but appreciable, increase in the levels of Guo, Cyd, and dThd was observed. In contrast to cell corpse phagocytosis, sheep red blood cell (SRBC) engulfment did not increase the amounts of nucleosides (Fig. 1 F and Fig. S3 F). As SRBCs do not have nuclei, nuclear DNA and RNA from cell corpses are likely to be the major sources of nucleosides accumulated in BM-Mphs. Nucleoside storage in pMphs and BM-Mphs suggested their engulfment of cell corpses during elicitation by thioglycolate in vivo or in vitro culture with M-CSF, respectively (Fig. S3, A and B).

We next studied nucleosides in lysosomal fractions and observed the significant accumulation of Guo, dGuo, Cyd, dCyd, and Ado in *Slc29a3*^−/−^ BM-Mphs (Fig. 1 G), suggesting lysosomal nucleoside accumulation in *Slc29a3*^−/−^ macrophages. We also found that both TLR7 and FLAG-tagged SLC29A3 were recruited to the cell corpse-containing phagosomes in the mouse macrophage cell line J774.1 (Fig. 1 H). These results suggest that SLC29A3 prevents TLR7 activation by exporting nucleosides from the compartment to which TLR7 is localized in WT macrophages but that SLC29A3 mutants fail to export nucleosides and thereby silence TLR7 in macrophages.

### TLR7 drives proliferation to increase phagocytes

**Figure 2.**
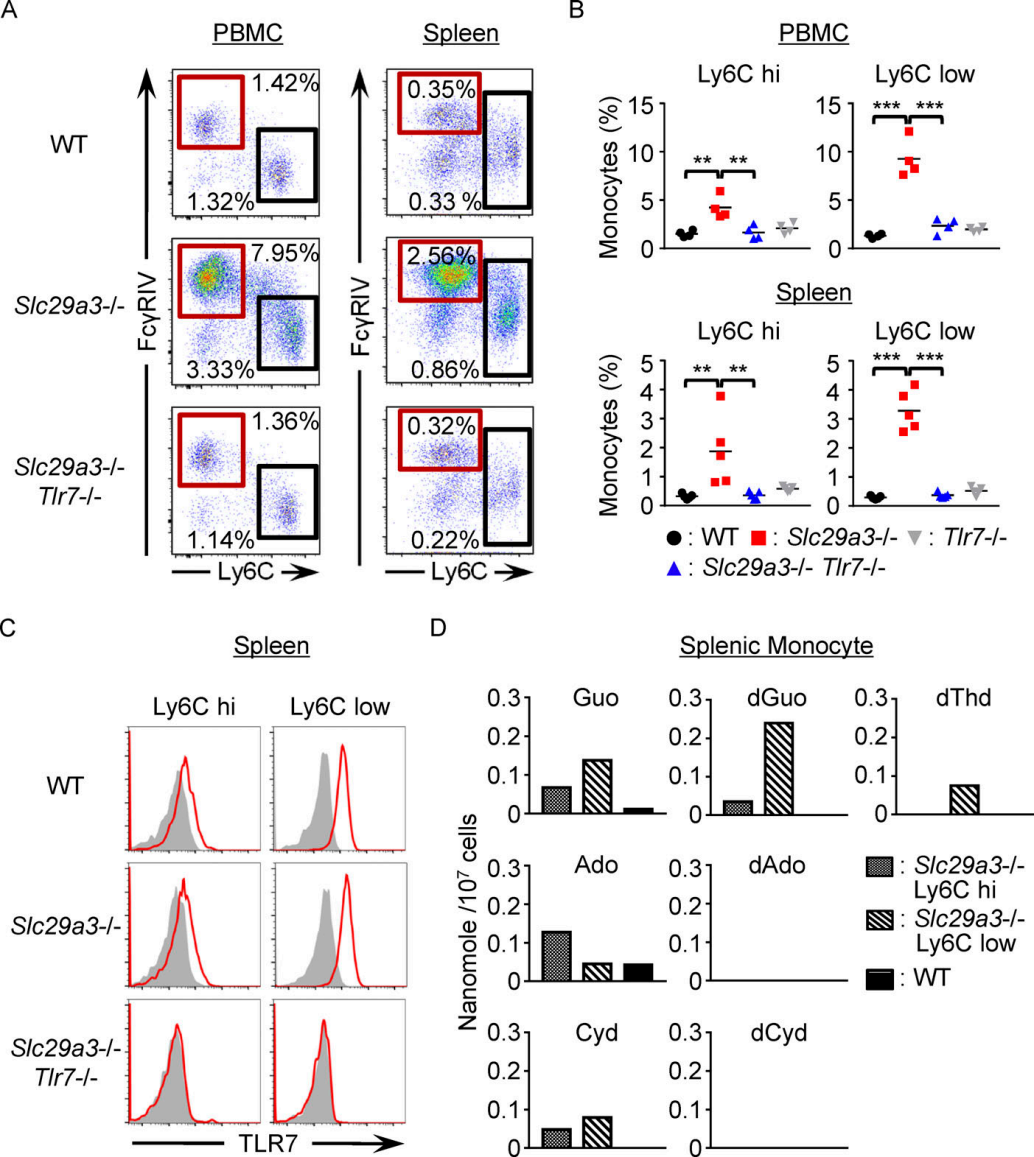
**Accumulated monocytes store nucleosides and express TLR7. (A)** Representative FACS analyses of CD11b^+^ Ly6G^–^ NK1.1^–^ CD11C^low^ IA/IE^low^ splenic and peripheral blood monocytes from WT, *Slc29a3*^−/−^, and *Slc29a3*^−/−^ *Tlr7*^−/−^ mice. The red and black squares show the gates of Ly6C^hi^ and Ly6C^low^ monocytes, respectively. **(B)** Dot plots show the percentages of Ly6C^low^ and Ly6C^hi^ monocytes in the peripheral blood (*n* = 4) and spleen (*n* = 5) from the indicated mice. **(C)** Red histograms show intracellular TLR7 expression levels in Ly6C^hi^ and Ly6C^low^ monocytes from the indicated mice. Gray histograms show staining with the isotype control antibodies. **(D)** Amounts (nanomole/10^7^ cells) of nucleosides in WT CD11b^+^ splenic monocytes or in Ly6C^hi^ and Ly6C^low^ splenic monocytes from *Slc29a3*^−/−^ mice. The experiments presented in D were performed twice and yielded the same results. The data shown in A and C are representative of at least three independent experiments. **P < 0.01 and ***P < 0.001.

Monocyte progenitors in the BM give rise to Ly6C^hi^ monocytes/macrophages, which mature into Ly6C^low^ monocytes/macrophages (Ginhoux and Jung, 2014). In the spleen and peripheral blood of the *Slc29a3*^‒/‒^ mice, both Ly6C^hi^ and Ly6C^low^ monocytes increased in a TLR7-dependent manner compared with those of WT and *Tlr7*^‒/‒^ mice (Fig. 2, A and B). Both Ly6C^hi^ and Ly6C^low^ splenic monocytes expressed TLR7 and SLC29A3 (Fig. 2 C) and stored nucleosides, such as Guo and dGuo (Fig. 2 D), suggesting that TLR7 is cell-autonomously activated in these subsets.

**Figure 3.**
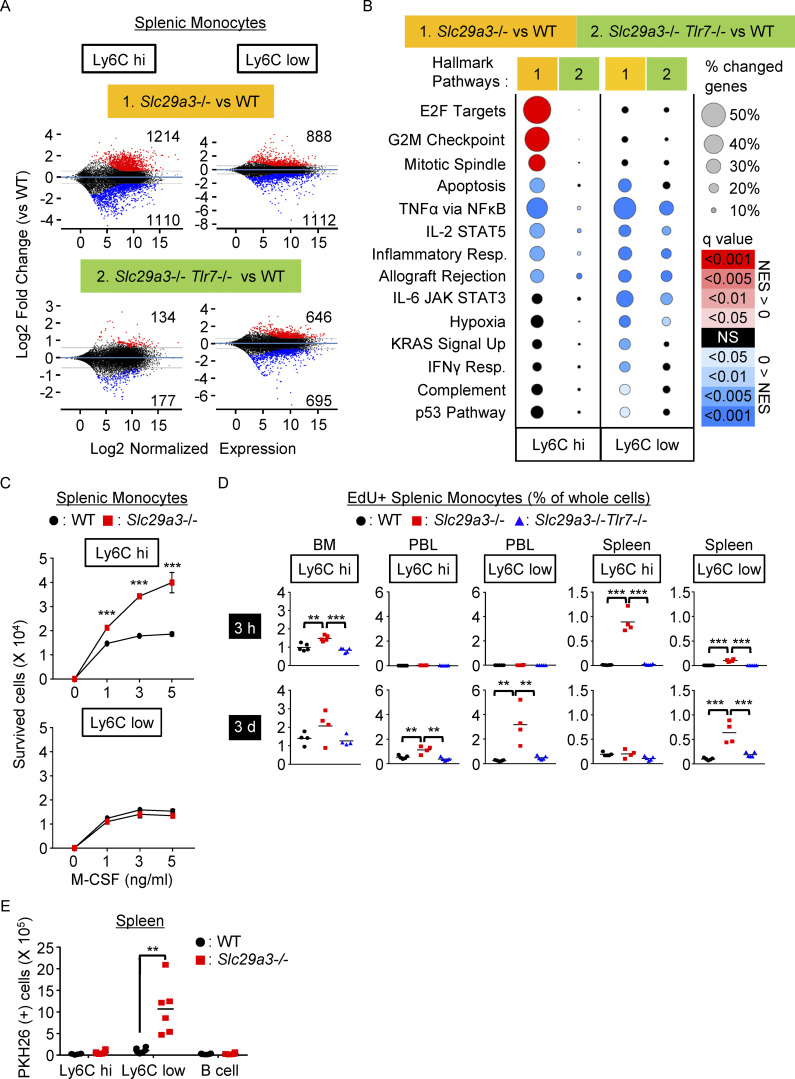
**Ly6C**^**hi**^ **monocytes proliferate to increase Ly6C**^**low**^ **phagocytes. (A)** Transcriptome analyses of Ly6C^hi^ and Ly6C^low^ monocytes from the spleen. MA-plots displaying log_2_ normalized expression (x axes) and log_2_ fold change of expression (y axes) for the comparisons of *Slc29a3*^−/−^ (*n* = 4) vs. WT (*n* = 4) monocytes and *Slc29a3*^−/−^ *Tlr7*^−/−^ (*n* = 4) vs. WT (*n* = 4) monocytes. More than 1.5-fold upregulated and downregulated genes are shown in red and blue, respectively. **(B)** GSEA of more than 1.5-fold changed genes in comparing Ly6C^hi^ and Ly6C^low^ splenic monocytes from *Slc29a3*^−/−^ and *Slc29a3*^−/−^ *Tlr7*^−/−^ mice with those from WT mice. Red and blue circles indicate positive and negative normalized enrichment scores (NES), respectively. Their sizes indicate the percentage of genes with a >1.5-fold change in each gene set. The color gradation indicates the *q* value of positive/negative enrichment. **(C)** Numbers of splenic Ly6C^hi^ and Ly6C^low^ monocytes from WT (black circle) and *Slc29a3*^−/−^ (red square) mice that survived for 4 d in vitro culture with M-CSF at the indicated concentrations. Mean values ± SD from triplicate samples are shown. The data are representative of three independent experiments. **(D)** Uptake of the thymidine analog EdU in vivo by monocytes from WT, *Slc29a3*^−/−^, and *Slc29a3*^−/−^ *Tlr7*^−/−^ mice (*n* = 4 or 5) at 3 h (upper) and 3 d (below) after intravenous EdU administration. Percentages of EdU^+^ cells in whole splenocytes are shown. **(E)** Number of PKH26^+^ cells in the indicated cell populations from the mice that had received PKH26-labelled dying thymocytes 1 h before analyses. Each dot represents the value for each mouse (*n* = 6). **P < 0.01 and ***P < 0.001.

To characterize TLR7 responses in these monocyte subsets, we performed transcriptome analyses comparing *Slc29a3*^−/−^ and *Slc29a3*^‒/‒^ *Tlr7*^‒/‒^ monocytes with WT monocytes. Gene expression in Ly6C^hi^ monocytes was TLR7-dependently changed (Fig. 3 A). Gene set enrichment analyses (GSEAs) of more than 1.5-fold altered genes revealed that proliferation-related gene sets such as “E2F targets,” “G2M checkpoint,” and “mitotic spindle” were positively enriched in *Slc29a3*^−/−^ Ly6C^hi^ monocytes (Fig. 3 B). These changes depended on TLR7 because such changes were not observed in *Slc29a3*^‒/‒^ *Tlr7*^‒/‒^ Ly6C^hi^ monocytes. To directly study the survival and proliferation of monocytes, splenic Ly6C^hi^ and Ly6C^low^ monocytes were sorted and cultured in vitro in the presence of M-CSF, which has been shown to promote histiocytosis in *Slc29a3*^−/−^ mice (Hsu et al., 2012). Ly6C^hi^, but not Ly6C^low^, monocytes from *Slc29a3*^−/−^ mice showed higher survival and proliferation in the presence of M-CSF at concentrations comparable with those in vivo (Fig. 3 C). As the cell surface expression of the M-CSF receptor CD115 was not appreciably upregulated in *Slc29a3*^−/−^ splenic monocytes (Fig. S3 G), augmented M-CSF responses are not explained by increased expression of cell surface CD115. The two signals via TLR7 or CD115 would synergistically drive the survival and proliferation of Ly6C^hi^ monocytes.

To study the in vivo proliferation of monocytes, mice were intravenously administered with the thymidine analog–EdU, and the percentages of EdU^+^ monocytes in the BM, peripheral blood, and spleen were analyzed 3 h after EdU administration. In WT mice, Edu^+^ proliferating monocytes were found only in BM Ly6C^hi^ monocytes (Fig. 3 D). The percentage of Edu^+^ monocytes in the BM TLR7-dependently increased in *Slc29a3*^−/−^ mice. Even more drastic changes were observed in the spleen, where Edu^+^ Ly6C^hi^ monocytes were found only in *Slc29a3*^−/−^ mice. We analyzed EdU^+^ monocytes 3 d after EdU administration and observed that the majority of Edu^+^ monocytes in the circulation and spleen turned Ly6C^low^ (Fig. 3 D), suggesting that proliferating Ly6C^hi^ monocytes mature into Ly6C^low^ monocytes within 3 d in the *Slc29a3*^−/−^ mice. Ly6C^low^ monocytes in the *Slc29a3*^*−/−*^ mice stored deoxyribonucleosides more than Ly6C^hi^ monocytes (Fig. 2 D). Because lysosomal deoxyribonucleosides were derived from cell corpses (Fig. 1, E and F; and Fig. S3, E and F), Ly6C^low^ monocytes are likely to have engulfed cell corpses during or after maturation to Ly6C^low^ monocytes. Consistent with this, splenic Ly6C^low^ monocytes in the *Slc29a3*^*−/−*^ mice engulfed intravenously administered dying thymocytes (Fig. 3 E). TLR7 in splenic Ly6C^low^ monocytes is likely to prolong survival because apoptotic-related gene sets are negatively enriched (Fig. 3 B). TLR7, therefore, increased the number of phagocytes in the *Slc29a3*^−/−^ mice. TLR7 might recognize lysosomal nucleoside storage as impaired phagocytosis and increase the phagocyte number as a compensatory mechanism.

### Mutually exclusive induction of proliferation and inflammation by TLR7

**Figure 4.**
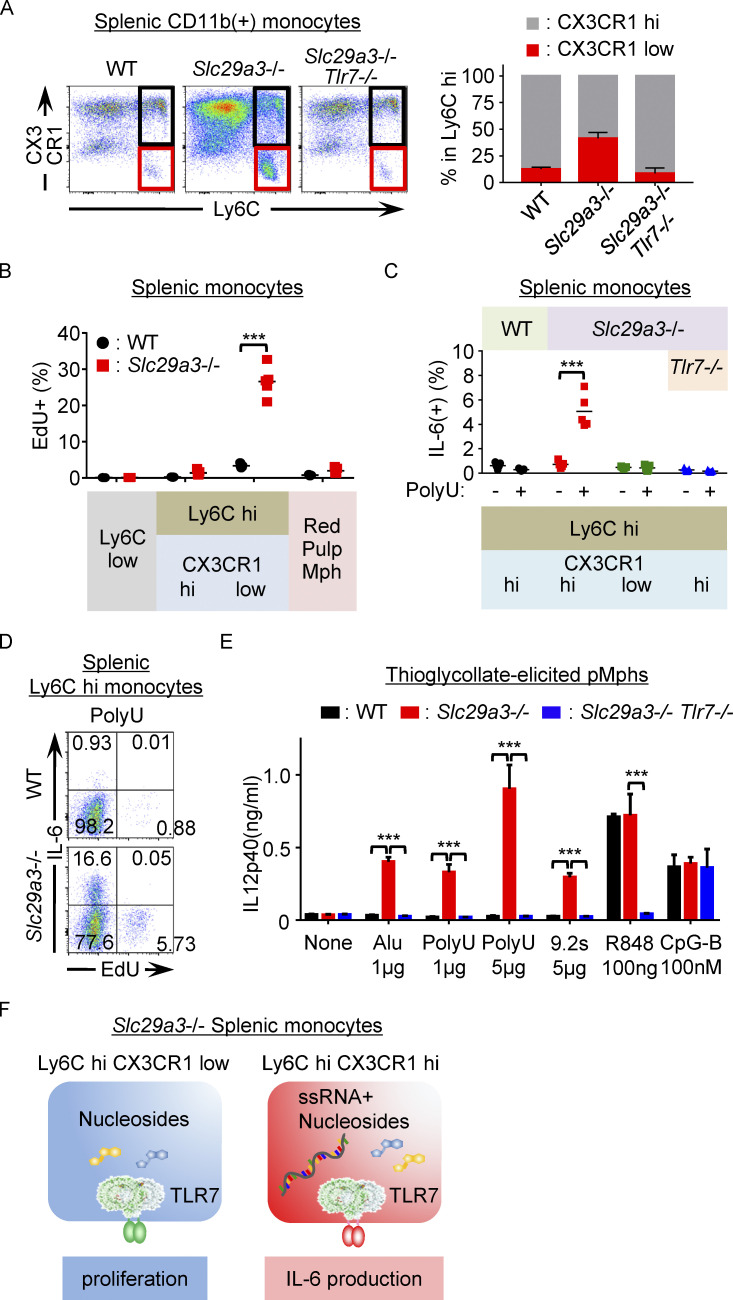
**TLR7 differentially induces proliferation and inflammation. (A)** Expression of CX3CR1 and Ly6C in splenic CD11b^+^ Ly6G^−^ NK1.1^−^ CD11C^low^ IA/IE^low^ monocytes from WT, *Slc29a3*^−/−^, and *Slc29a3*^−/−^ *Tlr7*^−/−^ mice (top). Black and red gates show Ly6C^hi^ CX3CR1^hi^ and Ly6C^hi^ CX3CR1^low^ monocytes, respectively. The right panel shows the percentages of the two Ly6C^hi^ monocyte subsets in the indicated mice (*n* = 4). **(B)** EdU uptake by each splenic monocyte subset during 1 h culture with 10 μM EdU. Each dot represents a value from a single mouse (*n* = 4). **(C)** Percentage of IL-6^+^ cells in each monocyte subset after in vitro stimulation with polyU (10 μg/ml) for 4 h. Brefeldin A (10 μg/ml) was added during cell stimulation. Each dot shows the values for each mouse from the indicated mice (*n* = 5). **(D)** Representative dot plot of EdU^+^ and IL-6^+^ cells in Ly6C^hi^ splenic monocytes treated with polyU + brefeldin A for 4 h. EdU was added during the last hour of stimulation. **(E)** IL-12 p40 production by thioglycolate-elicited pMphs after stimulation with TLR7 and TLR9 ligands for 18 h. Alu, Alu retroelements. The results are represented as mean values ± SD from triplicate samples. **(F)** Schematics showing the induction of two distinct TLR7 responses, proliferation, and IL-6 production, in *Slc29a3*^−/−^ mice. The data shown in D and E are representative of at least three independent experiments. ***P < 0.001.

To further narrow down the proliferating population of Ly6C^hi^ monocytes, we examined a marker specifically expressed or not expressed in proliferating Ly6C^hi^ monocytes. The cell surface expression of CX3CR1, a chemokine receptor for the membrane-tethered chemokine CX3CL1 (Landsman et al., 2009), increases with maturation from Ly6C^hi^ to Ly6C^low^ monocytes (Yona et al., 2013). We found that the CX3CR1^low^ population in Ly6C^hi^ monocytes significantly increased in a TLR7-dependent manner in the *Slc29a3*^−/−^ mice (Fig. 4 A). When splenic monocytes were cultured in vitro for 1 h with EdU, their uptake was detected in this immature monocyte subset but not in more mature Ly6C^hi^ CX3CR1^hi^ classical monocytes (Fig. 4 B). These results demonstrate that Ly6C^hi^ CX3CR1^low^ immature monocytes proliferate in response to lysosomal nucleoside storage in the *Slc29a3*^−/−^ mice.

**Figure S4.**
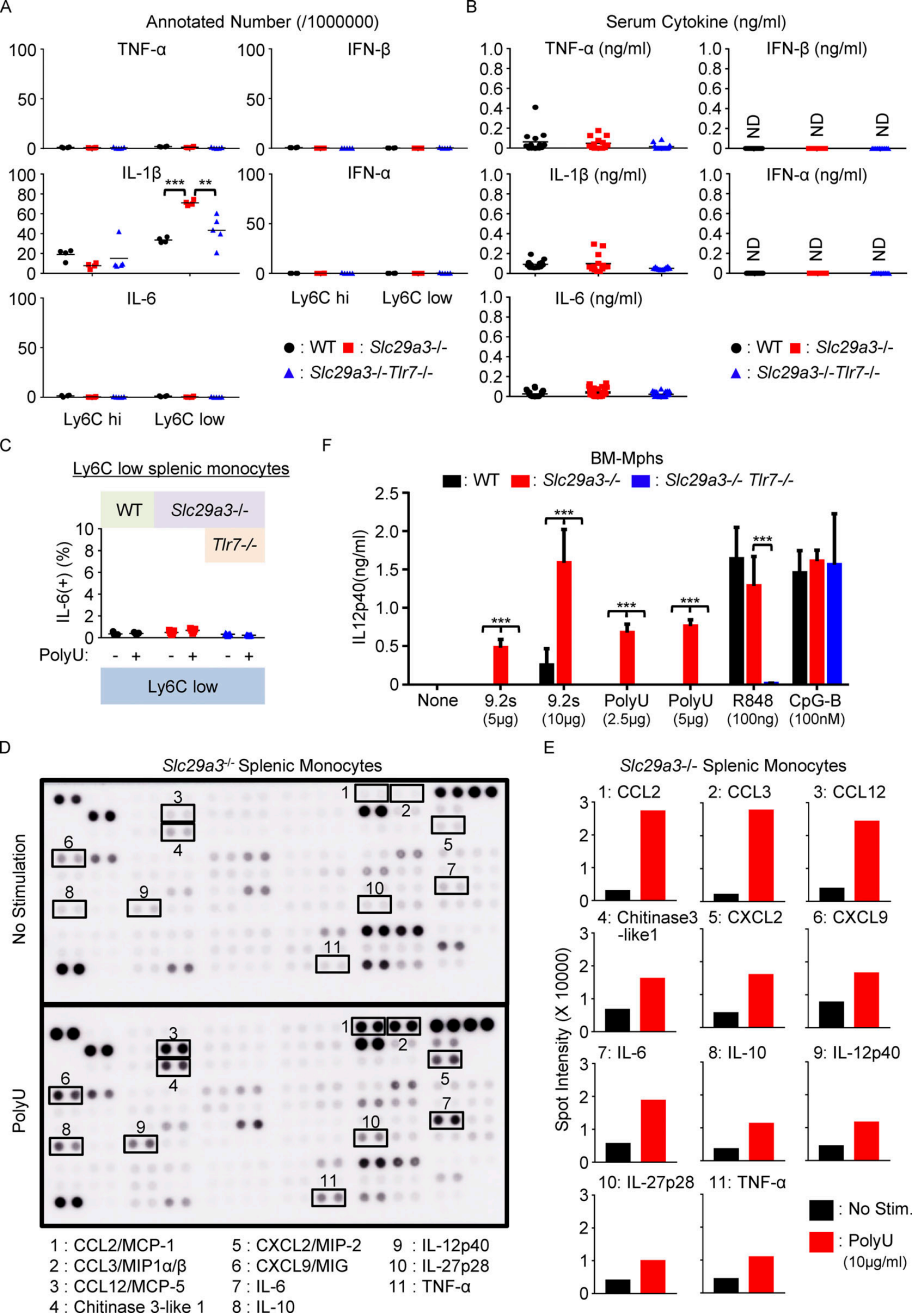
**Cytokine production in *Slc29a3***^**‒/‒**^ **mice. (A)** mRNA expression of cytokines in splenic Ly6C^hi^ and Ly6C^low^ monocytes from WT (black), *Slc29a3*^−/−^ (red), and *Slc29a3*^‒/‒^ *TLR7*^‒/‒^ (blue) mice. Each dot shows the normalized read count per 1 million reads from the RNA-seq analyses for each monocyte subset (*n* = 4). **(B)** Serum cytokine levels were determined using ELISA. Sera were collected from 4-to-6-mo old mice (*n* = 14). **P < 0.01 and ***P < 0.001. ND, not detected. **(C)** The percentage of IL-6^+^ cells in Ly6C^low^ Fcgr4^hi^ monocytes from indicated mice after in vitro stimulation with polyU (10 μg/ml) in the presence of brefeldin A (10 μg/ml) for 4 h. Each dot represents the value for each mouse (*n* = 5). **(D and E)** *Slc29a3*^‒/‒^ splenic monocytes were stimulated with polyU (10 μg/ml) for 18 h. Cytokines in the supernatants were detected using a cytokine antibody array (D). The results are shown as the mean signal intensity value (*n* = 2) for each cytokine spot (E). **(F)** IL-12 p40 production by BM-Mphs after stimulation with TLR7 and TLR9 ligands for 18 h. The results are represented as mean values ± SD from triplicate samples. *P < 0.05, **P < 0.01, and ***P < 0.001.

Because SLC29A3 disorders are considered to be inflammatory diseases in which IL-6 has pathogenic roles (Rafiq et al., 2017), we examined TLR7-dependent inflammatory responses in the *Slc29a3*^−/−^ mice. Unexpectedly, inflammation-associated gene sets such as “TNFα via NF-κB” and “inflammatory response” were negatively enriched in splenic Ly6C^hi^ monocytes in a manner dependent on TLR7 (Fig. 3 B). Consistent with this, SLC29A3 deficiency did not increase the expression levels of mRNAs encoding proinflammatory cytokines, such as IFN-α, IFN-β, IFN-γ, IL-6, IL-17A, IL-23, and TNF-α in Ly6C^hi^ and Ly6C^low^ monocytes (Fig. S4 A). Furthermore, proinflammatory cytokines, such as TNF-α, IFN-β, IL-1β, IFN-α, and IL-6, were not identifiable in the serum (Fig. S4 B). Considering that TLR7 responds to a combination of nucleosides and ORNs (Shibata et al., 2016; Zhang et al., 2016), these results suggest that ORNs do not accumulate and that nucleoside accumulation is not sufficient to induce TLR7-dependent inflammation in *Slc29a3*^−/−^ mice. We hypothesized that TLR7 response to ORN-generating ssRNA may be enhanced in nucleoside-laden *Slc29a3*^−/−^ monocytes. As predicted, *Slc29a3*^−/−^ Ly6C^hi^ CX3CR1^hi^ classical monocytes produced IL-6 upon polyU stimulation at 10 μg/ml, whereas WT Ly6C^hi^ monocytes did not respond to polyU (Fig. 4 C). In contrast, *Slc29a3*^−/−^ Ly6C^hi^ CX3CR1^low^ proliferating immature monocytes and Ly6C^low^ phagocytes did not respond to polyU treatment (Fig. 4 C and Fig. S4 C). Consistent with this finding, when splenic monocytes were treated with EdU and polyU, we only detected EdU^+^ or IL-6^+^ single-positive monocytes but not EdU^+^ IL-6^+^ double-positive monocytes (Fig. 4 D). These results suggest that TLR7 induces proliferation and inflammation in a mutually exclusive manner in immature and mature classical monocytes, respectively.

An antibody array for cytokines showed that polyU-stimulated splenic monocytes produced chemokines and cytokines, including CCL2, CCL3, CCL12, CXCL2, CXCL9, IL-6, TNF-α, IL-12p40, and IL-10, in *Slc29a3*^−/−^ mice (Fig. S4, D and E). Furthermore, *Slc29a3*^−/−^ professional macrophages, such as BM-Mphs and pMphs, exhibited a higher IL-12p40 production in response to polyU than did WT macrophages, whereas their responses to R848 and CpG-B were not altered (Fig. 4 E and Fig. S4 F). Inflammation in SLC29A3 disorders might be driven by an enhanced TLR7 response to ssRNA in Ly6C^hi^ splenic monocytes and peripheral macrophages. These results suggest that the TLR7 response to nucleoside storage varies with monocyte maturation from proliferation to an excessive inflammatory response to ssRNA (Fig. 4 F).

### FcRγ and DAP10 mediate TLR7 responses

**Figure 5.**
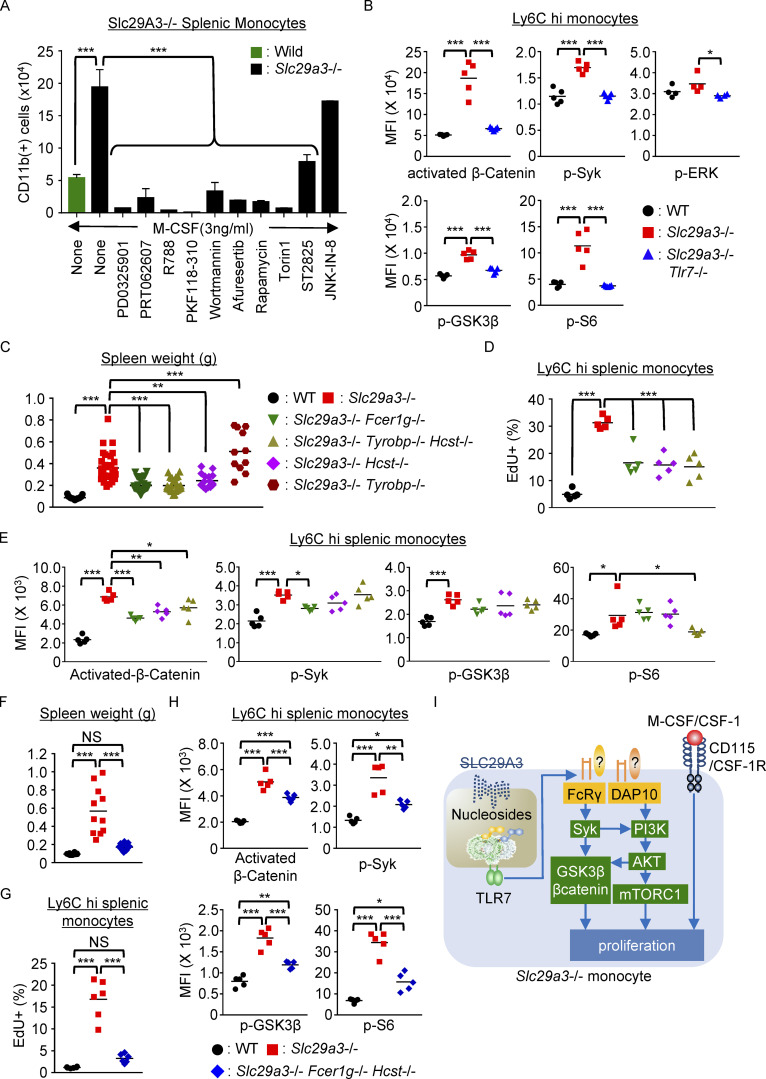
**FcRγ and DAP10 mediate TLR7-dependent proliferation in *Slc29a3***^**−/−**^ **mice. (A)** Number of splenic CD11b^+^ monocytes from WT (green) or *Slc29a3*^−/−^ (black) mice that survived in vitro 4-d culture in the presence of 3 ng/ml M-CSF under serum-free conditions. Cells were treated with inhibitors of MEK (PD0325901, 1 μM), Syk (PRT062007, 1 μM; R788, 0.5 μM), β-catenin (PKF118-120, 5 μM), PI3K (wortmannin, 10 μM), AKT (afuresertib, 5 μM), mTORC1 (rapamycin, 0.5 μM), mTORC1 and 2 (Torin1, 250 nM), MyD88 (ST2825, 10 μM), and JNK (JNK-IN-8, 1 μM). Bar graphs represent mean values ± SD from triplicate samples. The data are representative of three independent experiments. **(B, E, and H)** Mean fluorescence intensity (MFI) of staining with activation- and phospho-specific Abs to signaling molecules in Ly6C^hi^ monocytes from the indicated mice (*n* = 4–5). The colors of symbols in E denote mouse strains, as shown in C. **(C and F)** Spleen weight at 3–4 mo of age in the indicated mice (*n* = 11–32). *Fcer1g*, *Tyrobp*, and *Hcst* encode FcRγ, DAP12, and DAP10, respectively. **(D and G)** EdU uptake by CD11b^+^ Ly6G^–^ NK1.1^–^ CD11C^low^ IA/IE^low^ Ly6C^hi^ splenic monocytes after 1-h culture with EdU in vitro. Each dot shows the percentage of EdU^+^ cells in splenic Ly6C^hi^ monocytes from the indicated mice (*n* = 5). The colors of the symbols denote mouse strains, as shown in C or H. **(I)** The hypothetical model of TLR7-dependent proliferation in *Slc29a3*^−/−^ monocytes. NS, not significant. *P < 0.05, **P < 0.01, and ***P < 0.001.

Next, we focused on growth-promoting TLR7 signaling. We cultured whole splenocytes in vitro with 3 ng/ml M-CSF, where larger numbers of *Slc29a3*^‒/‒^ splenic monocytes survived than WT monocytes (Fig. 5 A). Inhibitors of MEK (PD0325901), Syk (PRT062607, R788), β-catenin (PKF118-120), PI3K (wortmannin), AKT (afuresertib), mTORC1 (rapamycin, Torin1), and MyD88 (ST2825), but not JNK (JNK-IN-8), reduced the number of surviving monocytes (Fig. 5 A). To examine the in vivo TLR7-dependent activation of these signaling molecules, flow cytometry analyses of Ly6C^hi^ monocytes were performed. Consistent with the effect of inhibitors, we found TLR7-dependent increases in the activated form of β-catenin and the phosphorylated forms of signaling molecules including Syk, GSK3β, and the ribosomal protein S6 (the mTORC1 downstream effector; Fig. 5 B). Unexpectedly, growth-promoting ERK phosphorylation was not altered by SLC29A3 deficiency. These results suggest that TLR7 activates signaling molecules such as Syk, GSK3β, β-catenin, and mTORC1 in *Slc29a3*^‒/‒^ Ly6C^hi^ monocytes.

**Figure S5.**
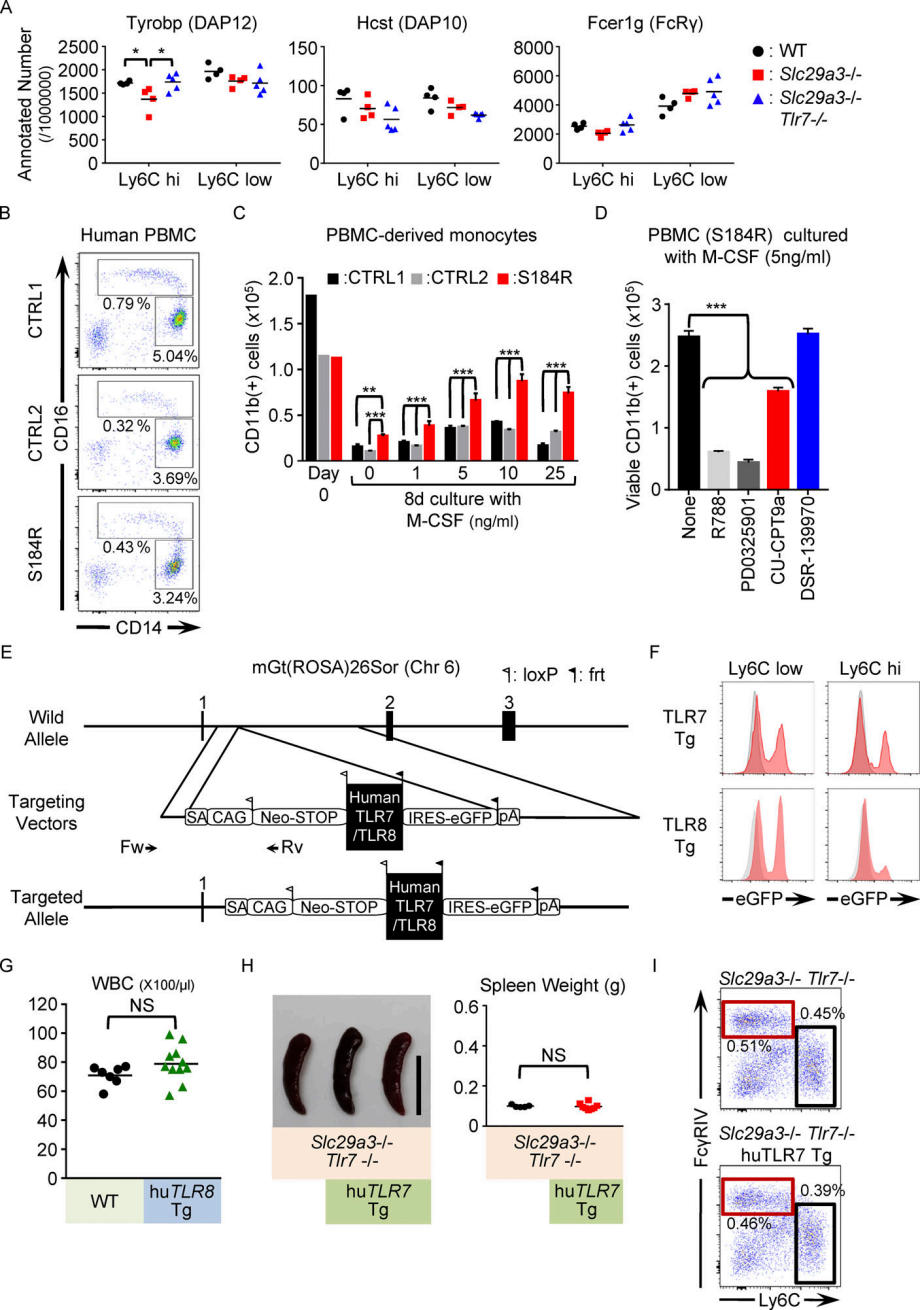
**TLR8 drives SLC29A3 disorders. (A)** mRNA expression of the ITAM adaptors in splenic Ly6C^hi^ and Ly6C^low^ monocytes from WT (black), *Slc29a3*^−/−^ (red), and *Slc29a3*^−/−^ *Tlr7*^−/−^ (blue) mice. Each dot represents the normalized read count per 1 million reads in the RNA-seq analyses (*n* = 4). **(B)** Expression of surface CD16/CD14 in HLA-DR^+^ CD15^−^ CD56^−^ PBMCs from the patient with the S184R *SLC29A3* mutation and from healthy subjects. **(C)** Numbers of CD11b^+^ CD15^−^ CD56^−^ monocytes in PBMCs from the patients (red) and healthy subjects (black and gray) that survived 4 d of culture with M-CSF at the indicated concentrations. **(D)** Number of CD11b^+^ cells that survived 8 d. PBMCs were cultured with 5 ng/ml M-CSF in the absence or presence of the indicated inhibitors of Syk (1 μM R788), MEK1/2 (1 μM PD0325901), TLR8 (10 μM CPT9a), and TLR7 (10 μM DSR-139970). **(E)** Schematic representation of the *Rosa26* locus, targeting vectors for the construction of human TLR7/8 transgenic mice, and targeted alleles. The targeting vectors contain both the neomycin resistance gene (Neo-STOP) flanked by loxP sites, human TLR7/8 cDNA, and the IRES-eGFP region flanked by frt sites. Cre recombinase removes floxed Neo-STOP (floxNeo) to initiate the expression of the transgene and eGFP. Arrows represent primer pairs for detecting homologous recombinant ES clones. SA, splice acceptor; CAG, CAG promoter; IRES, internal ribosome entry site; Neo-STOP, neomycin resistance gene; pA, polyadenylation signal. **(F)** Red histograms show eGFP expression of Ly6C^high^ and Ly6C^low^ monocytes in the spleen from huTLR7 or huTLR8 Tg mice (*Slc29a3*^‒/‒^ *Tlr7*^‒/‒^ *Rosa26* ^huTLR7/+^ *CAG*-Cre or *Slc29a3*^‒/‒^ *Tlr7*^‒/‒^ *Rosa26* ^huTLR8/+^ *Lyz2*-Cre mice). The expression level of eGFP was designed to reflect that of huTLR7 and huTLR8. Gray histograms represent background in control mice (*Slc29a3*^‒/‒^ *Tlr7*^‒/‒^ or *Slc29a3*^‒/‒^ *Tlr7*^‒/‒^ *Rosa26* ^huTLR8/+^ mice). **(G)** White blood cell (WBC) count in peripheral blood of WT or *Rosa26* ^huTLR8/+^ *Lyz2*-Cre (hu TLR8 Tg) mice. **(H)** Representative spleen images of 4–5-mo-old mice (left). Scale bar: 1 cm. The right panel shows spleen weights (*n* = 5–8) of *Slc29a3*^‒/‒^ *Tlr7*^‒/‒^ mice (CTRL) and *Slc29a3*^‒/‒^ *Tlr7*^‒/‒^ *Rosa26* ^huTLR7/+^ mice (hu TLR7-Tg). **(I)** Representative FACS analyses of CD11b^+^ Ly6G^‒^ NK1.1^‒^ CD11C^low^ IA/IE^low^ splenic monocytes from *Slc29a3*^‒/‒^ *Tlr7*^‒/‒^ or *Slc29a3*^‒/‒^ *Tlr7*^‒/‒^ *Rosa26* ^huTLR7/+^ mice. The red and black squares show the gates of the Ly6C^low^ and Ly6C^hi^ monocytes, respectively. The experiments using PBMCs from a patient with the S184R *SLC29A3* mutation (B–D) were conducted once. NS, not significant. *P < 0.05, **P < 0.01, and ***P < 0.001.

Because TLR7 primarily drives inflammation via NF-κB and IRF7 (Kawai and Akira, 2010), these growth-promoting signals might not be directly activated by TLR7. We hypothesized that TLR7 requires ITAM adaptors such as DAP12 and FcRγ to activate the growth-promoting signal because the ITAM adaptors are activated during TLR responses (Hamerman et al., 2009) and promote M-CSF-dependent macrophage proliferation by activating Syk, GSK3β, and β-catenin (Mócsai et al., 2010; Otero et al., 2009). Accumulated Ly6C^hi^ monocytes expressed mRNAs encoding DAP10, DAP12, and FcRγ (Fig. S5 A); therefore, we generated *Slc29a3*^‒/‒^ mice that lacked FcRγ, DAP10, DAP12, or DAP10+DAP12. Splenomegaly in *Slc29a3*^−/−^ mice was significantly ameliorated by the absence of FcRγ, DAP10, or DAP10+DAP12 (Fig. 5 C), whereas DAP12 deficiency significantly exacerbated splenomegaly in *Slc29a3*^‒/‒^ mice. Consistent with these results, EdU uptake by *Slc29a3*^‒/‒^ Ly6C^hi^ splenic monocytes was decreased by the lack of FcRγ, DAP10, or DAP10+DAP12 (Fig. 5 D). Next, we studied the activation status of β-catenin, Syk, GSK3β, and S6 in Ly6C^hi^ splenic monocytes (Fig. 5 E). The activated form of β-catenin was decreased by the lack of FcRγ, DAP10, or DAP10+DA12, whereas Syk phosphorylation was reduced only by FcRγ deficiency. Phosphorylation of GSK3β and S6 was not altered by single deletion of either FcRγ or DAP10 (Fig. 5 E). Finally, we generated *Slc29a3*^−/−^ mice lacking both FcRγ and DAP10 (*Slc29a3*^−/−^ *Hcst*^−/−^ *Fcer1g*^−/−^ mice), in which splenomegaly did not develop and splenic monocyte proliferation was reduced to the level of splenic monocytes of the WT mice (Fig. 5, F and G). TLR7-dependent activation of β-catenin and phosphorylation of Syk, GSK3β, and S6 in *Slc29a3*^−/−^ mice were all significantly reduced due to the lack of both FcRγ and DAP10 (Fig. 5 H). These results suggest that FcRγ and DAP10, and not DAP12, mediate growth-promoting TLR7 signals in *Slc29a3*^−/−^ mice (Fig. 5 I).

### TLR8-dependent histiocytosis in humans

**Figure 6.**
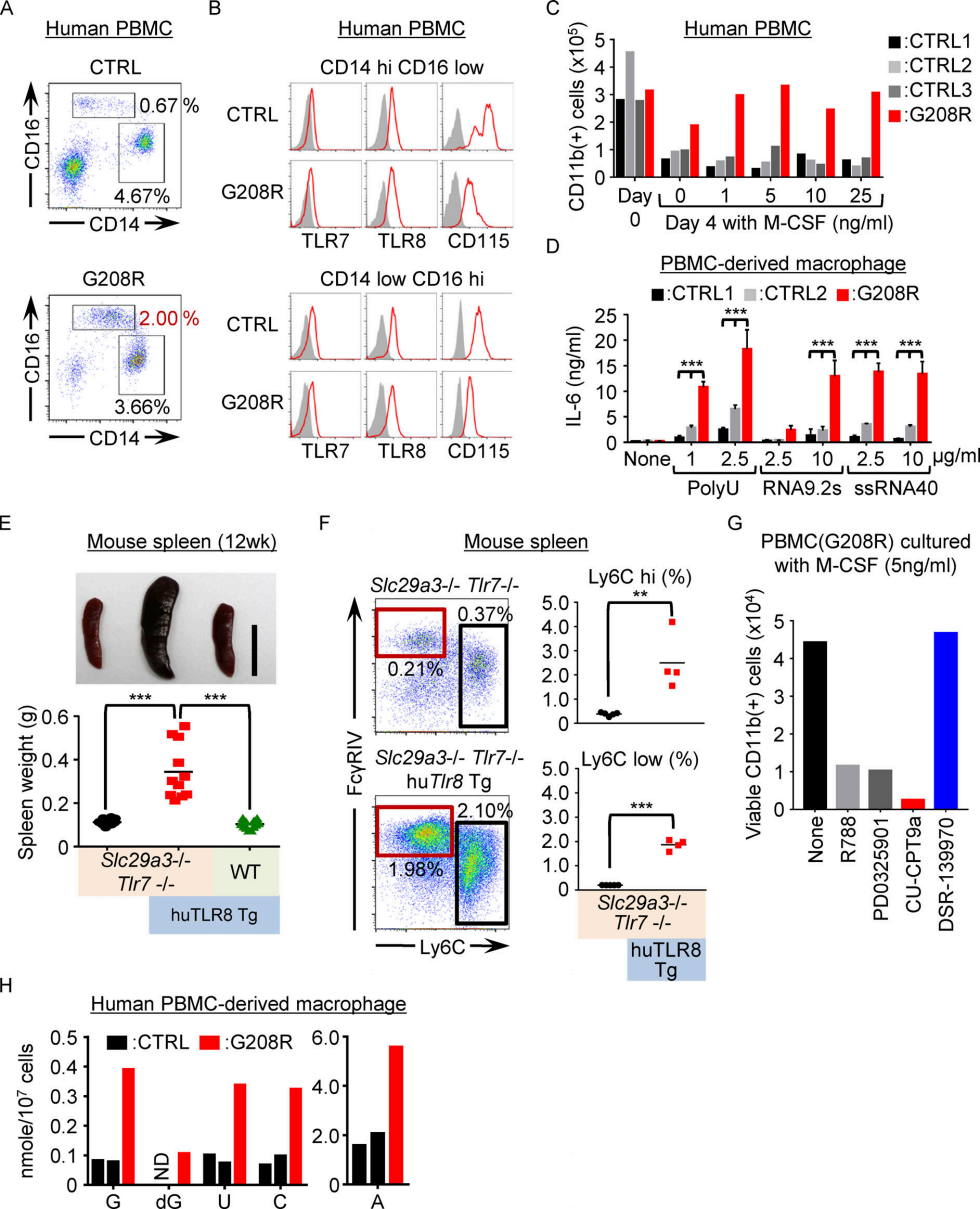
**TLR8 drives histiocytosis in SLC29A3 disorders. (A and B)** Expression of surface CD16/CD14/CD115 (A and B) and intracellular TLR7/TlR8 (B) in HLA-DR^+^ CD15^−^ CD56^−^ PBMCs from a patient with the G208R *SLC29A3* mutation and a healthy subject. Red and gray histograms show staining with the indicated and isotype-matched control antibodies, respectively. **(C)** The number of surviving CD11b^+^ CD15^−^ CD56^−^ monocytes in PBMCs from patients (red) or healthy subjects (black and gray) after 4 d of culture with M-CSF at the indicated concentrations. **(D)** PBMC-derived macrophages from the patient (red) or two control subjects (black and gray) were stimulated with the indicated TLR7 or TLR8 ssRNA ligands at the indicated concentrations. IL-6 production was evaluated using ELISA. The bars represent mean values ± SD from triplicate samples. **(E)** Representative spleen images of 3-mo-old mice (top). Scale bar, 1 cm. The bottom panel shows spleen weight (*n* = 10–14) of the indicated mice: *Slc29a3*^−/−^ *Tlr7*^−/−^ *Rosa26* ^huTLR8/+^ (black, *Slc29a3*^−/−^ *Tlr7*^−/−^); *Slc29a3*^−/−^ *Tlr7*^−/−^ *Rosa26* ^huTLR8/+^ *Lyz2*-Cre (red, *Slc29a3*^−/−^ *Tlr7*^−/−^ huTLR8 Tg), and *Rosa26* ^huTLR8/+^ *Lyz2*-Cre mice (green, huTLR8 Tg). **(F)** Representative FACS analyses of CD11b^+^ Ly6G^−^ NK1.1^−^ CD11C^low^ IA/IE^low^ splenic monocytes from *Slc29a3*^−/−^ *Tlr7*^−/−^ *Rosa26* ^huTLR8/+^ (*Slc29a3*^‒/‒^ *Tlr7*^‒/‒^) or *Slc29a3*^‒/‒^ *Tlr7*^‒/‒^ *Rosa26* ^huTLR8/+^ *Lyz2*-Cre(*Slc29a3*^‒/‒^ *Tlr7*^‒/‒^ huTLR8 Tg) mice. Red and black squares show the gates of Ly6C^low^ and Ly6C^hi^ monocytes, respectively. Dot plots show the percentages of Ly6C^low^ and Ly6C^hi^ monocytes in the spleen of the indicated mice (*n* = 4). **(G)** CD11b^+^ cells that survived in vitro PBMC culture with 5 ng/ml M-CSF in the presence of the indicated inhibitors of Syk (1 μM R788), MEK1/2 (1 μM PD0325901), TLR8 (10 μM CPT9a), and TLR7 (10 μM DSR-139970). **(H)** The amounts (nanomole/10^7^ cells) of nucleosides accumulated in PBMC-derived macrophages. The experiments presented in A–D, G, and H were performed twice and yielded the same results. ND, not detectable. **P < 0.01 and ***P < 0.001.

Finally, we investigated whether human monocytes from SLC29A3 disorders show enhanced TLR7/8 responses. In human peripheral blood mononuclear cells (PBMCs) from a patient harboring the *SLC29A3* p.Gly208Arg (G208R) mutation (Fujita et al., 2015), CD14^low^ CD16^hi^ monocytes, which are equivalent to the mouse Ly6C^low^ monocytes (Cros et al., 2010; Ginhoux and Jung, 2014), were increased by approximately threefold (Fig. 6 A). The expression levels of TLR7 and TLR8 in CD14^hi^ CD16^low^ and CD14^low^ CD16^hi^ monocytes were not altered in the patient (Fig. 6 B). To study monocyte proliferation and survival, PBMCs were cultured in vitro with human M-CSF. Despite lower expression of surface CD115, a larger number of HLA-DR^+^ CD11b^+^ monocytes from the patient survived in vitro culture than those from three healthy subjects (Fig. 6 C). PBMCs were allowed to differentiate into macrophages by human M-CSF and IL-4, and they were stimulated with the TLR7/8 ligands polyU and RNA9.2S, or TLR8 ligand ssRNA40 (Shibata et al., 2016). Macrophages harboring the G208R *SLC29A3* mutation produced larger amounts of IL-6 than the control macrophages in response to TLR7/8 and TLR8 ssRNA ligands, strongly suggesting that the *SLC29A3* mutation enhanced TLR7 and TLR8 responses to ssRNA in macrophages (Fig. 6 D). We also examined PBMCs from another patient harboring the *SLC29A3* p.Ser184Arg (S184R) mutation (Ramot et al., 2010). The percentage of monocytes did not increase (Fig. S5 B); however, proliferation and survival in vitro in the presence of M-CSF were significantly enhanced (Fig. S5 C). These results demonstrate that the phenotypes in the *Slc29a3*^−/−^ mice were consistent with those of the patients with SLC29A3 mutation.

To determine whether TLR8 drives histiocytosis in humans, huTLR8 was expressed in Ly6C^hi^ and Ly6C^low^ monocytes by Lyz2-Cre drivers (*Rosa26*^huTLR8/+^/*Lyz2*-Cre mice; Fig. S5, E and F). Although human TLR8 expression under the control of its regulatory regions drives wasting diseases with leukocytopenia (Guiducci et al., 2013), *Lyz2*-dependent TLR8 expression, specifically in monocytes, caused neither leukocytopenia nor splenomegaly in WT mice (Fig. 6 E and Fig. S5 G) but drove monocytosis and splenomegaly in *Slc29a3*^‒/‒^ *Tlr7*^−/−^ mice (Fig. 6, E and F), suggesting that human TLR8 substituted for mouse TLR7 in *Slc29a3*^−/−^ monocytes. In contrast, broad human TLR7 expression under the CAG-Cre driver did not drive splenomegaly in WT and *Slc29a3*^−/−^ *Tlr7*^−/−^ mice (Fig. S5, H and I). Consistent with these results in mice, the TLR8 antagonist CU-CPT9a (Zhang et al., 2018), but not the TLR7 antagonist DSR-139970 (Cpd-7; Tojo et al., 2020), inhibited the in vitro survival of human macrophages harboring G208R or S184R SLC29A3 mutation (Fig. 6 G and Fig. S5 D). Furthermore, Syk and MEK inhibitors, R788 and PD0325901, also showed an inhibitory effect, suggesting that Syk and MEK mediate growth-promoting TLR8 signal in human SLC29A3 disorders. Additionally, we detected approximately threefold higher amounts of Urd, the TLR8 ligand (Heil et al., 2004; Shibata et al., 2016; Tanji et al., 2015), in macrophages harboring the G208R *SLC29A3* mutation than in those from healthy subjects (Fig. 6 H). These results strongly suggest that TLR8 drives histiocytosis in SLC29A3 disorders.

## Discussion

In this study, we identified a previously unknown histiocytosis-driving program that is activated by germline loss-of-function mutations in *SLC29A3*. Nucleosides, including Cyd, Guo, and dGuo, consistently accumulate in lysates of the spleen, splenic macrophages, BM-Mphs, and pMphs. We confirmed the accumulation of these nucleosides in the endosomal compartments of BM-Mphs. As TLR7 and SCL29A3 are both localized to the endosomal compartments, SLC29A3 deficiency results in the accumulation of the TLR7 ligands, Guo, and dGuo, in TLR7-containing compartments, and thereby TLR7 activation drives histiocytosis in *Slc29a3*^−/−^ mice. Two lines of evidence support the possibility that TLR7 and TLR8 drive histiocytosis in a cell-autonomous manner. First, sorted Ly6C^hi^ monocytes showed enhanced proliferation/survival in vitro. Second, macrophage-specific expression of human TLR8 by the *Lyz2*-Cre driver could drive histiocytosis in *Slc29a3*^*−/−*^ *TLR7*^*−/−*^ mice. However, a specific TLR7 depletion in macrophages would be required to formally prove that TLR7 in macrophages drives histiocytosis in *Slc29a3*^*−/−*^ mice.

Mouse TLR8 does not respond to nucleosides and ssRNAs (Heil et al., 2004) and negatively regulates TLR7 (Demaria et al., 2010). However, TLR8 deficiency did not alter histiocytosis in *Slc29a3*^*−/−*^ mice. In humans, TLR8 responds to Urd- and purine-containing ORNs (Shibata et al., 2016; Tanji et al., 2015; Zhang et al., 2016). TLR8 drove histiocytosis in *Slc29a3*^*−/−*^ *Tlr7*^*−/−*^ mice and a TLR8 antagonist inhibited the survival of *SLC29A3*^*G208R/G208R*^ monocytes in vitro. We detected Urd accumulation in human peripheral blood. These results suggest that human TLR8 drives histiocytosis in SLC29A3 disorders. However, we need to be careful about the results using human samples because we only studied two patients. This study needs to be confirmed with additional samples from SLC29A3 disorder patients.

Both TLR7 and TLR8 were expressed in CD14^hi^ CD16^low^ classical and CD14^low^ CD16^hi^ patrolling monocytes. As TLR8 was more highly expressed in these monocytes than TLR7, TLR8 might be preferentially activated in peripheral blood monocytes in SLC29A3 disorders. However, TLR7 may be more highly expressed and could drive proliferation in certain tissue-resident macrophages. The role of human TLR7 in SLC29A3 disorders remains unclear.

SLC29A3 disorders cause various manifestations such as hyperpigmented, hypertrichotic cutaneous patches, hepatosplenomegaly, cardiac anomalies, sensorineural hearing loss, and short stature (Molho-Pessach et al., 2014; Morgan et al., 2010). These systemic manifestations suggest that macrophages accumulate in multiple organs. In *Slc29a3*^*−/−*^ mice, macrophages increased in various organs including the spleen, liver, pancreas, and kidneys in a TLR7-dependent manner. However, macrophages accumulated predominantly in the spleen. The spleen is mainly populated by BM-derived monocytes/macrophages, whereas other organs largely contain tissue-resident macrophages, which are derived from yolk sac macrophages and self-replicate (Ginhoux and Guilliams, 2016). Like splenic macrophages of WT mice, splenic histiocytes originate from the BM (Hsu et al., 2012). Continuous histiocyte supply from the BM may be the reason why histiocytosis was primarily found in the spleen of *Slc29a3*^*−/−*^ mice.

TLR7 and TLR8 are activated by a combination of nucleosides and ORNs (Shibata et al., 2016; Tanji et al., 2015; Zhang et al., 2016). While we observed that TLR7/8-activating nucleosides such as Guo, dGuo, and Urd accumulate in human and mouse macrophages, we have not examined ORNs. In contrast to nucleosides, ORN detection was difficult owing to their sequence length and complexity. Therefore, we could only perform functional studies. We found that polyU-dependent IL-6 production was enhanced in *Slc29a3*^−/−^ monocytes/macrophages. PolyU degradation increases ORN concentration but does not impact Guo in endosomal compartments, suggesting that increased ORNs are sufficient to activate TLR7 in Guo/dGuo-laden *Slc29a3*^−/−^ monocytes/macrophages but not in WT macrophages. If both ORNs and nucleosides accumulate due to SLC29A3 deficiency, TLR7 would be constitutively activated to produce proinflammatory cytokines in *Slc29a3*^−/−^ monocytes/macrophages. This was not the case, strongly suggesting that ORNs do not accumulate in *Slc29a3*^−/−^ monocytes/macrophages.

In contrast to inflammatory responses, nucleoside accumulation appeared sufficient to drive monocyte/macrophage proliferation, suggesting that TLR7-dependent proliferation is distinct from inflammatory responses. These two TLR7 responses were activated in a mutually exclusive manner. Proliferating immature monocytes did not show polyU-dependent IL-6 production, whereas mature monocytes did produce IL-6 upon polyU stimulation but did not proliferate in *Slc29a3*^−/−^ mice. The molecular difference between these two TLR7 responses might be explained by FcRγ and DAP10 because both were required for monocyte/macrophage proliferation in *Slc29a3*^−/−^ mice. In contrast, FcRγ negatively regulates TLR-dependent proinflammatory cytokine production in macrophages and dendritic cells (Hamerman et al., 2009). These adaptors may play a role in the mutually exclusive induction of inflammation and proliferation in monocytes/macrophages. Mechanisms by which FcRγ and DAP10 work downstream of TLR7 remain unclarified. As FcRγ associates with the IL-3 receptor to promote cytokine production (Hida et al., 2009), FcRγ and DAP10 might directly associate with TLR7 to drive proliferation. Alternatively, these adaptors might associate with FcRs and the TREM family of receptors, which work downstream of TLR7 to promote macrophage proliferation.

In contrast to monocytes/macrophages, B cell, and pDC numbers were not increased in *Slc29a3*^−/−^ mice, despite their expression of TLR7. Their differing behavior from monocytes/macrophages can be explained in part by phagocytic activity. B cells and pDCs are less phagocytic than monocytes/macrophages (Aderem and Underhill, 1999; Dalgaard et al., 2005) and thus store much smaller amounts of nucleoside. Another and probably more important difference is FcRγ expression in monocytes/macrophages. As FcRγ is required for TLR7-dependent proliferation, FcRγ-negative B cells and pDCs would fail to proliferate even if nucleoside accumulates. In human SLC29A3 disorders, monocyte/macrophage proliferation is likely driven by TLR8. As TLR8 is predominantly expressed in monocytes/macrophages but not in B cells or pDCs, TLR8 activation may explain monocyte/macrophage-restricted accumulation in human SLC29A3 disorders.

Constitutive TLR7 activation in mice by small chemical ligand administration, transgenic TLR7 expression, and Unc93B1 mutation drives monocytosis and splenomegaly (Akilesh et al., 2019; Deane et al., 2007; Fukui et al., 2011). Furthermore, TLR7-dependent monocytosis occurs in lupus-prone NZBWF1 mice (Murakami et al., 2021). Therefore, constitutive TLR7 activation generally drives histiocytosis/monocytosis in mice. Much less is known about histiocytosis by constitutive human TLR8 activation. *TLR8* gain-of-function mutations drive splenomegaly, which might be due to macrophage accumulation in the spleen, although monocytosis in peripheral blood is not apparent (Aluri et al., 2021). Macrophage responses to accumulated nucleosides are reminiscent of macrophage responses to heme following erythrocyte phagocytosis. Heme stress in macrophages induces their differentiation into red pulp macrophages, which are phagocytes specialized for red blood cell clearance (Haldar et al., 2014). We would like to refer to metabolite-dependent macrophage proliferation and maturation as the lysosomal stress response. TLR7 and TLR8 serve as metabolite sensors to activate lysosomal stress responses, which drive histiocytosis unless the stress is relieved. These results demonstrate that SLC29A3 disorders are lysosomal stress diseases.

## Materials and methods

### Generation of *Slc29a3*^−/−^, *Tlr7*^−/−^, and *Tlr8*^−/−^ mice

C57BL/6 *Slc29a3*^−/−^ and *Tlr7*^−/−^ mice were generated using the CRISPR/CAS9 system. gRNA target sites on *Slc29a3* and *Tlr7* were determined using CRISPRdirect software (http://crispr.dbcls.jp/), and 5′-AGC​TTC​TTG​ATG​GTT​ACT​CG-3′ and 5′-GAA​CAG​TTG​GCC​AAT​CTC​TC-3′ sequences were chosen for the construction of *Slc29a3*^−/−^ and *Tlr7*^−/−^ mice, respectively. These gRNA target sequences were cloned into the BbsI site of the pKLV-U6 gRNA (BbsI)-PGKpuro2ABFP vector (Addgene plasmid 50946). Using the constructed vectors as templates, gRNAs were synthesized by in vitro transcription using the MEGA shortscript T7 Transcription Kit (Thermo Fisher Scientific). Additionally, hCAS9 mRNA was synthesized from the hCAS9 sequence in pX458 (Addgene plasmid 48138) in vitro using the mMESSAGE mMACHIN T7 ULTRA Transcription Kit (Thermo Fisher Scientific). To generate Slc29a3^−/−^ Tlr7^−/−^ mice, synthesized gRNAs (50 ng/μl) targeting the *Slc29a3* and *Tlr7* genes, hCAS9 mRNA (100 ng/μl), and the repair donor DNA (100 ng/μl) to introduce a stop codon into the Slc29a3 *locus* (5′-tgc​ctc​tga​gga​caa​tgt​ata​cca​cag​ctc​caa​tgc​tgt​cta​cag​agc​ccT​GAT​AGC​GTA​AAG​CAC​TGA​GGA​AGc​gag​taa​cca​tca​aga​agc​tga​cca​gga​agc​cct​gct​ggg​gaa​act​act​a-3′; lowercase and capital letters represent homology arm and insertion sequence, respectively) were injected into zygotes from C57BL/6 mice at the pronuclei stage. The injected zygotes were then transferred to the oviducts of pseudopregnant female C57BL/6 mice. Candidates for *Slc29a3*^−/−^ *Tlr7*^−/−^ mice were typed by PCR using primer pairs for *Slc29a3* (Fw: 5′-CCA​GCA​TGG​ACG​AGA​GAT​GTC​TTC-3′, Rv: 5′-GCA​CCA​TTG​AAG​CGA​TCC​TCT​GG-3′) and *Tlr7* (Fw: 5′-GAG​GGT​ATG​CCG​CCA​AAT​CTA​AAG​AAT​C-3′, Rv: 5′-CTG​ATG​TCT​AGA​TAG​CGC​AAT​TGC-3′). The PCR products were analyzed using the MCE-202 MultiNA Microchip Electrophoresis System for DNA/RNA Analysis (SHIMAZU), and *Slc29a3*^−/−^ *Tlr7*^−/−^ male mice were chosen for mating with WT C57BL/6 female mice. *Slc29a3*^+/−^ *Tlr7*^+/−^ mice were mated to establish *Slc29a3*^−/−^ and *Slc29a3*^−/−^ *Tlr7*^−/−^ mice. The sequences of the mutated allele in *Slc29a3*^−/−^ and *Tlr7*^−/−^ mice were confirmed by direct sequencing performed by FASMAC.

E14.1 embryonic stem (ES) cells were subjected to transfection with the vectors designed to target the *Tlr8* locus (Fig. S2 J). Clones demonstrating resistance to G418 and ganciclovir were selected, and homologous recombination was verified by PCR and further confirmed using Southern blot analysis. Targeted ES clones were then injected into blastocysts derived from C57BL/6 mice to generate chimeric mice, which were subsequently bred to obtain *Tlr8*^−/−^ mice. The *Tlr8*^−/−^ mice were identified through PCR typing utilizing specific primers (Fw: 5′-TCC​TTA​GGA​AAA​CAT​GCC​CCC​TCA​GTC-3′, WT Rv: 5′-GTC​TGT​TGA​GAG​AGG​TTT​CCG​AAG​ACG-3′, Mut Rv: 5′-ATC​GCC​TTC​TAT​CGC​CTT​CTT​GAC​GAG-3′)

### Generation of TLR7/TLR8 transgenic mice

Cre-inducible human TLR7 (huTLR7) or human TLR8 (huTLR8) transgenic mice (Tg mice) were generated and backcrossed with C57BL/6 background *Tlr7*^−/−^ *SLC29A3*^−/−^ mice to evaluate the functions of huTLR7 and huTLR8 in vivo. *Rosa26* locus on mouse chromosome6 was targeted to construct Cre-inducible human TLR7/8 knock-in Tg mice. Specifically, huTLR7 or huTLR8 coding sequences were cloned into the CAG-STOP-eGFP-ROSA26TV/CTV targeting vector, which was a kind gift from Yoshiteru Sasaki (Tohoku Medical and Pharmaceutical University, Sendai, Japan; #15912; Addgene). These targeting vectors were electroporated into C57BL/6N background JM8.A3N1 ES cells, and neomycin-resistant ES clones were screened by PCR using 5′ external Fw (5′-TCC​TCA​GAG​AGC​CTC​GGC​TAG​GTA​G-3′) and Neo Rv (5′-AAT​GGC​CGC​TTT​TCT​GGA​TTC​ATC-3′); then, homologous recombinants were injected into C57/BL6N derived blastocysts to generate chimeric mice. Chimeric mice were mated with C57BL/6 mice to obtain *Rosa26*^floxSTOPhuTLR7/+^ and *Rosa26*^floxSTOPhuTLR8/+^ mice. *Rosa26*^*f*loxSTOPhuTLR7/+^ mice were crossed with CAG-Cre Tg mice to generate *Rosa26*^huTLR7/+^(huTLR7 Tg). Since Rosa26^floxSTOPhuTLR8/+^/CAG-Cre mice showed an embryonic lethal phenotype, they were crossed with Lyz2-Cre Tg mice to obtain *Rosa26*^huTLR8/+^/Lyz2-Cre (huTLR8 Tg), which showed normal growth and development under specific pathogen-free (SPF) conditions. Finally, huTLR7 Tg and huTLR8 Tg mice were crossed with *Slc29a3*^−/−^ *Tlr7*^−/−^ mice. Generated *Slc29a3*^−/−^ *Tlr7*^−/−^ *Rosa26* ^huTLR7/+^ and *Slc29a3*^−/−^ *Tlr7*^−/−^ *Rosa26* ^floxSTOPhuTLR8/+^ Lyz2-Cre mice were subjected to experiments.

### Mice

WT C57BL/6 mice were purchased from Japan SLC, Inc. C57BL/6 *Tlr8*^−/−^, *FcRγ*^−/−^, *Hcst* (DAP10)^−/−^, *Tyrobp* (DAP12)^−/−^, and *Hcst*^−/−^ *Tyrobp*^−/−^ mice were previously reported (Gilfillan et al., 2002; Inui et al., 2009; Koga et al., 2004). All animals were housed in SPF facilities at the Institute of Medical Science, University of Tokyo (IMSUT). All animal experiments were approved by the Institutional Animal Care and Use Committee of the IMSUT.

### Reagents

PD0325901 (mirdametinib), PRT062607 (P505-15) HCl, R788 (fostamatinib), wortmannin (KY 12420), afuresertib (GSK2110183), AY-22989 (Rapamycin), and JNK-IN-8 were purchased from Selleck Chemical. PKF118-310 was purchased from Sigma-Aldrich. Torin 1 and ST2825 were purchased from Calbiochem/Merck Millipore and ChemScence, respectively.

Stable isotope-labeled nucleosides, G (13C10, 98%; 15N5, 96–98%), U (13C9, 98%; 15N2, 96–98%), A (13C10, 98%; 15N5, 96–98%), C (13C9, 98%; 15N3, 96–98%), and dG (13C10, 98%; 15N5, 96–98%) were purchased from Cambridge Isotope Laboratories for the quantification of nucleosides by LC-MS/MS. The EdU used in the in vivo proliferation assay was purchased from Tokyo Chemical Industry Co.

DSR-139970 (Cpd7), a TLR7 inhibitor, was kindly provided by Sumitomo Pharma Co., Ltd. CU-CPT9a, a specific TLR8 inhibitor, and R848 were purchased from InvivoGen. SRBC used in the phagocytic assay were obtained from Cosmo Bio Co.

RNA9.2s (20mer, UsGsUsCsCsUsUsCsAsAsUsGsUsCsCsUsUsCsAsA), ssRNA40 (GsCsCsCsGsUsCsUsGsUsUsGsUsGsUsGsAsCsUsC), PolyU (19mer, UsUsUsUsUsUsUsUsUsUsUsUsUsUsUsUsUsUsU), and ODN1668 (20mer, dTsdCsdCsdAsdTsdGsdAsdCsdGsdTsdTsdCsdCsTdsdGsdAsdTsdGsdCsdT), in which “s” depicts a phosphothioate linkage, were synthesized by FASMAC.

### Establishment of anti-human TLR7/8 mAbs

To establish an anti-human TLR7/8 mAb, WT Wistar rats and BALB/C mice were immunized several times with purified huTLR7/8 ectodomain and Ba/F3 cells expressing huTLR7/8 mixed with TiterMax Gold. 4 d after final immunization, splenocytes and SP2/O myeloma cells were fused with polyethylene glycol. After selection by hypoxanthine/aminopterin/thymidine (HAT), antibodies against TLR7 and TLR8 were selected by flow cytometry analyses using Ba/F3 cells expressing huTLR7/8. Anti-huTLR7 and huTLR8 mAbs were designated as rE3 (rat IgG2a/κ) and M7B (mouse IgG1/κ), respectively. The purity of the mAbs was checked by SDS-PAGE and Coomassie brilliant blue staining, and biotinylated mAbs were used for subsequent experiments.

### Flow cytometry

For the preparation of samples for flow cytometry analyses, the spleens were minced using glass slides and the BM cells were pipetted several times to disperse the cells in RPMI 1640 culture medium. The suspended samples were teased using nylon mesh to remove tissue debris. All samples were treated with BD Pharm lysing buffer (BD Biosciences) to remove red blood cells before being subjected to cell staining. Cell surface staining for flow cytometry analyses was performed using fluorescence-activated cell sorting (FACS) staining buffer (1× PBS with 2.5% FBS and 0.1% NaN_3_). The prepared cell samples were incubated for 10 min with an unconjugated anti-mouse CD16/32 blocking mAb (clone 95) to prevent nonspecific staining in the staining buffer. The cell samples were then stained with fluorescein-conjugated mAbs for 15 min on ice.

PBMCs, BM cells, and splenocytes from mice were stained with fluorescent dye–conjugated mAbs specific for the following markers after blocking antibody treatment: CD11b (clone M1/70), FcγRIV (clone 9E9), CD3ε (clone 145-2c11), CD19 (clone 6D5), CD11c (clone N418), CD71 (clone R17217), Ter119/Erythroid cells (clone Ter119), Ly6C (clone HK1.4), and Ly6G (clone 1A8).

Human PBMCs were assessed using fluorescent dye–conjugated antibodies specific for the following markers: HLA-DR (clone G46-6), CD14 (clone M5E2), CD16 (clone 3G8), CD15 (clone HI98), CD56 (clone 5.1H11), CD3ε (clone SK7), and CD19 (clone HIB19). These antibodies were purchased from eBioscience, BioLegend, BD Biosciences, and TONBO Biosciences. Biotinylated anti-mouse TLR7 (A94B10) mAb was previously established in our laboratory (Kanno et al., 2015).

To detect endolysosomal TLR7 and TLR8, cells, after cell surface staining, were fixed and permeabilized using a Fixation/Permeabilization Solution Kit (BD Biosciences) and stained again with biotinylated anti-mouse/human TLR7/8 mAb and PE streptavidin (BioLegend). To detect intracellular mouse IL-6 in splenocytes stimulated with various ligands in the presence of Brefeldin A (10 μg/ml), cells, after cell surface staining, were fixed with Fixation Buffer (BioLegend), permeabilized using 1× Click-iT, saponin-based permeabilization, and wash reagent (Invitrogen Thermo Fisher Scientific), and then stained with PE-conjugated anti-mouse IL-6 mAb (clone MP5-20F3; BioLegend). Stained cells were analyzed using a BD LSR Fortessa cell analyzer (BD Biosciences) or an ID7000 Spectral Cell Analyzer (Sony Biotechnology). All flow data were analyzed using FlowJo software v10.7 (BD Biosciences).

### Intracellular phospho-flow cytometry

Phosphorylation of signaling molecules was detected by flow cytometry. First, 3–4 × 10^6^ splenocytes after cell surface staining were fixed with Cyto-Fast Fix/Perm buffer (BioLegend) for 20 min at room temperature and washed twice with FACS staining buffer. Fixed cells were further permeabilized by adding prechilled True-Phos Perm Buffer (BioLegend) and incubated at −20°C for 2–3 h. After washing twice with FACS staining buffer, permeabilized cells were stained with PE-conjugated anti-p-Syk (clone C87C1; 1:50 dilution; Cell Signaling Technology), anti-p-S6 (clone D57.2.2E; 1:100 dilution; Cell Signaling Technology), anti-p-GSK3β (clone D85E12; 1:50 dilution; Cell Signaling Technology), anti-p44/42 MAPK (Erk1/2; clone 137F5; 1:50 dilution; Cell Signaling Technology), or Fluor647-conjugated anti-active β-catenin (clone 8E7; 1:400 dilution; Merck Millipore) and subjected to flow cytometry analyses.

### Cell sorting of pDCs and splenic monocytes

Cell staining for sorting was performed in a sorting buffer (1× PBS with 10% FBS, 10 mM HEPES, and 1 mM sodium pyruvate). Flt3L-induced BM cells were stained with anti-CD11c/B220 mAbs, and CD11c^+^B220^+^ cells were sorted as BM-derived pDCs. For purification of Ly6Clow and Ly6C^high^ splenic monocytes, whole splenocytes from WT and *Slc29a3*^−/−^ mice were sequentially incubated with biotinylated anti-mouse CD3 (clone 145-2C11)/CD19 (clone 6D5)/NK1.1 (clone PK136)/Ly6G (clone aA8)/TER-119/erythroid cells (clone Ter-119) and Streptavidin MicroBeads (Miltenyi Biotec). The magnetically labeled cells were removed using autoMACS (Miltenyi Biotec), and the enriched cells were stained with anti-mouse CD11b/Ly6C/Fcgr4/NK1.1/Ly6G/Siglec-F mAb. Ly6C^low^Fcgr4^high^ and Ly6C^high^Fcgr4^low^ in CD11b^+^NK1.1^−^Ly6G cell subsets were sorted as Ly6C^low^ and Ly6C^high^ monocytes, respectively. Cell sorting was performed using a FACS ARIA III cell sorter (BD Biosciences).

### RNA-seq analysis

Ly6C^low^ and Ly6C^high^ splenic monocytes were obtained by FACS sorting from WT, *Slc29a3*^−/−^, and *Slc29a3*^−/−^ *Tlr7*^−/−^ mice. Total RNA was extracted using RNeasy Mini Kits (Qiagen), and the quality of RNA was evaluated using the Agilent Bioanalyzer device (Agilent Technologies). The samples with RIN (RNA integrity number) value of more than 7.3 were subjected to library preparation. RNA-seq libraries were prepared with 1 ng of total RNA using an Ion AmpliSeq Transcriptome Mouse Gene Expression kit (Thermo Fisher Scientific) according to the manufacturer’s instructions. The libraries were sequenced on Ion Proton using an Ion PI Hi-Q Sequencing 200 kit and Ion PI Chip v3 (Thermo Fisher Scientific). The FASTQ files were generated using AmpliSeqRNA plug-in v5.2.0.3 in the Torrent Suite Software v5.2.2 (Thermo Fisher Scientific) and analyzed by ROSALIND (https://rosalind.bio/, OnRamp Bioinformatics), which is a cloud-based bioinformatics software. Raw reads were trimmed using Cutadapt, and quality scores were assessed using FastQC2. Reads were aligned to the *Mus musculus* genome build mm10 using the STAR aligner. Individual sample reads were quantified using HTseq and normalized via relative log expression (RLE) using the DESeq2 R library. DEseq2 was used to determine the fold changes and P values. Genes showing more than a 1.5-fold change in expression (P < 0.05) were considered to be significantly altered. To interpret gene expression profiles, GSEA was performed using MSigDB hallmark gene sets to explore the pathways associated with SLC29A3 deficiency. Enriched pathways with false discovery rate–adjusted P values lower than 0.05 are shown in Fig. 3 B.

### EdU proliferation assay

In vivo and in vitro proliferation assays were performed using a Click-iT Plus EdU Alexa Fluor 488 Flow Cytometry Assay Kit (Invitrogen) according to the manufacturer’s instructions. In brief, mice were injected intravenously with 1 mg 5-ethynyl-2′-deoxyuridine (EdU) dissolved in 1× PBS. Spleen and blood samples were collected at 3 h or 3 d after injection. Then, erythrocytes were completely lysed by BD Pharm Lyse lysing buffer (BD Biosciences) to collect splenocytes and PBMCs. After blocking splenocytes and PBMCs with anti-CD16/32 (clone:95) mAb, the samples were stained with fluorescent dye–conjugated mAbs. The stained samples were then fixed with BD Cytofix (BD Biosciences) and permeabilized using 1× Click-iT saponin-based permeabilization and washing reagent. Finally, EdU incorporated into the genomic DNA was stained using Click-iT EdU reaction cocktails. EdU-positive cells were detected using a BD LSR Fortessa cell analyzer (BD Biosciences) or a spectral flow cytometer ID7000 (Sony Biotechnology).

### Proliferation assay in vitro

Proliferation assays were performed in serum-free AIM-V medium (Thermo Fisher Scientific) supplemented with penicillin-streptomycin-glutamine (Thermo Fisher Scientific). Whole mouse splenocytes and human PBMCs were plated at a density of 5 × 10^6^ cells per well in a Cepallet W-type 24-well microplate (DIC) and cultured for 4 d with or without mouse/human M-CSF (Peprotech). Surviving macrophages that adhered to 24-well plates were detached by lowering the temperature on ice. The collected cells were incubated with both LIVE/DEAD fixable aqua fluorescent reactive dye (Invitrogen) and SYTOX Green dead cell stain (Invitrogen) in 1× PBS for dead cell staining. Whole mouse splenocytes and human PBMCs were stained with CD11b/Ly6G/NK1.1/Ly6C/Fcgr4 and CD11b/HLA-DR/CD14/CD16 after blocking antibody treatment, respectively. The number of live macrophages was estimated using flow-count fluorospheres (Beckman Coulter) and flow cytometry.

Sorted Ly6C^low^ and Ly6C^high^ monocytes were plated on 96-well plates (BD Falcon) at 2 × 10^4^ cells/well and cultured for 4 d with or without mouse M-CSF. The surviving macrophages were detected by the CellTiter-Glo 2.0 Cell Viability Assay (Promega) following the manufacturer’s protocol and the macrophage number was estimated by comparing with FACS analyses to the control samples whose monocyte numbers were counted.

### LC-MS analysis

Quantitative nucleoside analyses were performed using an LC-MS system equipped with a reversed-phase column (2.0 mm I.D. × 100 mm) packed with Develosil C30 UG (3 μm particle, Nomura Chemical) connected to a hybrid quadrupole-orbitrap mass spectrometer (Q Exactive, Thermo Fisher Scientific) through an electrospray interface. For analyses of nucleoside accumulation in mouse cells and human PBMC-derived macrophages, 1 × 10^7^ cells were lysed using 400 μl D solution (7 M guanidine hydrochloride and 0.5 M Tris-HCl/10 mM EDTANa_2_, pH 8.5) containing stable isotope-labeled nucleosides (final standard nucleoside concentration; 1 nmol/400 μl of A/U/G/C/dG). For the analyses of nucleoside accumulation in tissues, 100 mg of each tissue was lysed with 400 μl D solution containing stable isotope-labeled nucleosides (final standard nucleoside concentration: 1 nmol/400 μl of C/dG, 10 nmol/400 μl of U/G, and 100 nmol/400 μl of A). The extract was centrifuged at 10,000 ×*g* for 30 min and the supernatant was diluted 40- to 200-fold with 10 mM ammonium acetate buffer (pH 6.0). Samples (∼1–100 pmol nucleosides/40 μl) were loaded into a reversed-phase column and eluted with a 30 min linear gradient from 2 to 12% acetonitrile in 10 mM ammonium acetate buffer (pH 6.0) at a flow rate of 100 μl/min. The eluate from the first 6 min was automatically wasted by switching a three-way electric valve to remove guanidine hydrochloride from the system and was subsequently sprayed into a mass spectrometer at 3.0 kV operating in positive-ion mode. Mass spectra were acquired at a resolution of 35,000 from m/z 200 to 305. Each nucleoside in the sample cells or tissues was quantified from the peak height relative to that of the corresponding isotope-labeled standard nucleoside. All LC-MS data were processed and analyzed using Xcalibur (version 3.0.63, Thermo Fisher Scientific) and Excel 2013 (Microsoft).

### Lysosome isolation

Lysosomes were isolated from BM-Mphs using Lysosome Enrichment Kit for tissues and cultured cells (Thermo Fisher Scientific) In brief, 1 × 10^8^ BM-Mphs from WT or *Slc29a3*^−/−^ mice in 800 μl Lysosome Enrichment Reagent A were homogenized on ice by passing through a 0.5-ml insulin syringe with 29G needle (TERUMO). Cell lysates mixed with 800 μl Lysosome Enrichment Reagent B were then centrifuged at 500 ×*g* for 10 min at 4°C and supernatants were subjected to gradient centrifugation. In ultracentrifuge tubes, the discontinuous density gradient was prepared according to the manufacturer’s instructions and the lysate containing 15% OptiPrep Media was overlayed on top of the density gradients. After ultracentrifugation of the samples at 145,000 ×*g* for 2 h at 4°C by Himac CS100FNX (Hitachi), the top band containing isolated lysosomes was collected and centrifuged after dilution by three volumes of 1× PBS at 18,000 ×*g* for 30 min at 4°C to make the pellet. Collected lysosome pellets were lysed with 50 μl D solution containing stable isotope-labeled nucleosides and subjected to LC-MS analyses.

### Platelet and cell counts

Platelet numbers in PBMCs were analyzed using an automatic hematology analyzer (Celltac α; Nihon Kohden). Cell number was measured using an automated cell counter, CellDrop BF (DeNovix).

### Preparation of splenic B cells

Splenic B cells were purified by negative selection using CD43 MicroBeads (Miltenyi Biotec). Splenocytes from WT and Slc29A3^−/−^ mice were labeled with CD43 magnetic beads and CD43-negative splenic B cells were enriched using autoMACS (Miltenyi Biotec) and subjected to experiments.

### Preparation of BM-derived macrophages and pDCs

BM cells were collected from the tibiae, femora, and pelvises of WT, *Slc29a3*^−/−^, and *Slc29a3*^−/−^*Tlr7*^−/−^ mice, and red blood cells were removed using BD Pharm Lyse lysing buffer. For the preparation of BM-Mphs, BM cells were plated at a density of 7 × 10^6^ cells per well on a non-tissue culture polystyrene 94-mm petri dish (Greiner Bio-One) and cultured in 10 ml RPMI medium (Gibco Thermo Fisher Scientific) supplemented with 10% FBS, penicillin-streptomycin-glutamine (Gibco Thermo Fisher Scientific), 50 μM 2-ME, and 100 ng/ml recombinant murine macrophage colony-stimulating factor (M-CSF; PeproTech, Inc.) for 6 d. The attached cells on petri dishes were collected and used as BM-Mphs. For BM-pDCs, BM cells were plated at a density of 2.5 × 10^7^ cells per 10-cm cell culture dish (Greiner Bio-One) and cultured in 10 ml RPMI 1640 medium (Gibco) supplemented with 10% FBS, penicillin-streptomycin-glutamine, 50 μM 2-ME, and 100 ng/ml recombinant murine FMS-like tyrosine kinase-3 ligand (Flt3L, PeproTech, Inc.) for 7 d. Flt3L-induced pDCs were stained with anti-CD11c/B220 mAbs, and CD11c^+^B220^+^ cells were sorted as BM-pDCs using a FACSAria flow cytometer (BD Biosciences).

### Preparation of human PBMCs and macrophages

All experiments using human samples were approved by the Institutional Ethics Review Boards of the IMSUT, Jichi Medical University, and Hiroshima University.

To prepare human peripheral blood mononuclear cells (hPBMCs), 7 ml of EDTA-anticoagulated whole blood was treated with 45 ml BD Pharm lysis buffer (BD Biosciences) to completely lyse red blood cells. hPBMCs collected after centrifugation were subjected to FACS analyses and survival assays or allowed to differentiate into macrophages. To induce human macrophages, hPBMCs were plated in 94 × 16 mm petri dishes (Greiner) at a density of 1.0 × 10^7^ cells per dish and cultured in 10 ml RPMI 1640 medium (Gibco) supplemented with 10% FBS, penicillin-streptomycin-glutamine (Gibco), 50 μM 2-ME, 100 ng/ml of recombinant human M-CSF (PeproTech, Inc.), and 20 ng of recombinant human IL-4 (PeproTech, Inc.) for 7 d. After removing floating cells with 1× PBS, the attached cells were collected as human macrophages and subjected to LC-MS and ELISA.

### Cytokine measurements by ELISA

Mouse thioglycollate-elicited PECs and mouse BM-Mphs were cultured in flat-bottom 96-well plates (BD Falcon) at 1 × 10^5^ cells/well. Human PBMC-derived macrophages were cultured in flat-bottomed 96-well plates at 1 × 10^4^ cells/well. All types of immune cells were stimulated with the indicated ligands for 16–20 h and cytokine concentrations in the supernatant were measured using ELISA. Serum cytokine concentrations in the mice were measured using ELISA. The concentrations of mouse IL-12p40, mouse TNF-α, mouse IL-1β, mouse IL-6, and human TNF-α in the supernatant were measured using Ready-Set-Go! ELISA kits (eBioscience). Mouse IFN-α and IFN-β concentrations in the supernatant were measured using IFN-α/β ELISA kits (PBL Assay Science).

### Cytokine antibody array

The production of 111 cytokines by splenic monocytes was quantified using the Proteome Profiler Mouse XL Cytokine Array (R&D Systems). Cytokine antibody array was performed according to the manufacturer’s instructions. In brief, the antibody array membrane was incubated with 200 μl culture supernatant of splenic monocytes overnight at 4°C. After incubation with the samples, the membranes were sequentially treated with a detection antibody cocktail and streptavidin-HRP. Finally, the membranes were treated with ECL Select Western Blotting Detection Reagent (GE Healthcare), and the chemiluminescent signal on the membranes was detected using an ImageQuant LAS 500 imager system (GE Healthcare). The intensity of each spot was quantified using the Quick Spots image analysis software (Western Vision Software).

After the cytokine antibody array, IL-6 production by the splenic monocyte subsets was further determined by flow cytometry to confirm the results from the Proteome Profiler Antibody Arrays.

### Cell death induction and phagocytosis assay in vivo

Cell death was induced by treatment of thymocytes at 47°C for 20 min, and then, cells were incubated at 37°C for 3 h before subjecting the cell corpses to the phagocytosis assay. Before the phagocytosis assay in vivo, cell corpses were stained with the PKH26 Red Fluorescent Cell Linker Kit for General Cell Membrane Labeling (Sigma-Aldrich) according to the manufacturer’s instructions. Then, 5 × 10^7^ PKH26-stained cell corpses were intravenously administered to mice, and mouse spleens were collected 2 h after cell corpse administration. Immune cells engulfing PKH26-positive cell corpses were detected using flow cytometry.

### Lentiviral transduction

FLAG-tagged human SLC29A3 was expressed in the mouse macrophage cell line J774.1 cells using lentiviral transduction. The cDNA of FLAG-SLC29A3 was substituted with the BFP2Apuro sequence in the lentiviral pKLV-U6gRNA(BbsI)-PGKpuro2ABFP vector (plasmid 50946; Addgene), excluding the U6gRNA(BbsI) site. The ViraPower Lentiviral expression system (Thermo Fisher Scientific) was used to prepare the lentivirus for Flag-SLC29A3 overexpression according to the manufacturer’s instructions. Supernatants containing lentivirus particles were collected 24 h after transfection and used for transduction.

### Structured illumination microscopy

Macrophages from the J774.1 cell line were allowed to adhere to collagen-coated coverslips overnight and were stimulated by 1 mM PMA for 2 h. Cells attached to coverslips were treated with the heat-treated dying thymocytes for 1 h. After engulfment, the cells were fixed with 4% paraformaldehyde for 10 min and then permeabilized with 1× PBS containing 0.2% saponin for 30 min. After blocking with 2.5% BSA Blocking One (Nacalai Tesque) for 30 min, cells were incubated with anti-TLR7 antibody and anti-HA antibody (Roche) at 37°C for 90 min.

After washing the cells three times, the cells were incubated for 90 min at 37°C with Alexa Fluor 488–conjugated goat anti-mouse and Alexa Fluor 568 goat anti-rat antibodies and DAPI (Invitrogen). Fluorescence microscopy was performed using a Nikon Structured illumination microscope (N-SIM, Nikon) at excitation wavelengths of 405, 488, and 561 nm with a CFI Apochromat TIRF 100× objective lens (1.49 NA, NIKON). Data acquisition was performed in 3D SIM mode before the image reconstruction using NIS-Element software. Each image represents more than three independent experiments.

### NF-κB–dependent luciferase reporter assay

HEK293T cells were cultured in RPMI 1640 (Nakarai Tesque), supplemented with 10% FBS, 2 mM L-glutamine (Gibco), and 50 μM 2-mercaptoethanol. To evaluate the activity of mouse or human TLR7, HEK293T cells were seeded onto collagen-coated 6-well plates at a density of 1 × 10^6^ cells per well and transiently transfected with WT mouse or human TLR7 cDNA in pMX-puro-IRES-rat CD2 (kindly provided by Prof. Kitamura, University of Tokyo, Tokyo, Japan; 1 μg), along with WT human Unc93B1 cDNA in pMX-puro (1 μg) and a pNL3.2.NF-κB-RE[NlucP/NF-κB-RE/Hygro] vector (25 ng), using PEI (polyethylenimine “Max,” MW 40,000; Polysciences, Inc.) at 24 h prior to stimulation. After 20 h of transfection, cells were reseeded onto collagen-coated flat 96-well plates (Corning) at a density of 1 × 10^5^ cells per well. Following a preculture for 4 h, the attached cells were stimulated with various ligands in the presence of 20 μl/ml DOTAP for 6 h. Subsequently, the Nanoluc activity in the stimulated cells was quantified using the Nano-Glo Luciferase assay system (Promega). Finally, the relative light unit (RLU) of bioluminescence was measured using GloMax Explorer (Promega).

### Statistical analysis

Statistical significance between the two groups was determined using a two-tailed, unpaired *t* test with Holm–Sidak correction. To determine significant differences between more than three groups, one-way ANOVA followed by Dunnett’s multiple comparison test was employed in this study. All data are represented as the mean ± SD and graphs were made using PRISM. Statistical significance was set at P < 0.05. *P < 0.05, **P < 0.01, and ***P < 0.001.

### Online supplemental material

Fig. S1 shows the strategy for generating *Slc29a3*^‒/‒^*Tlr7*^‒/‒^ mice, and mouse or human TLR7 responses to various nucleoside ligands (related to Fig. 1). Fig. S2 shows the TLR7-dependent phenotypes in *Slc29a3*^−/−^ mice, the strategy for generation of *Tlr8*^−/−^ mice, and the phenotypes observed in *Slc29a3*^‒/‒^*Tlr8*^‒/‒^ mice (related to Fig. 1). Fig. S3 shows nucleoside accumulation in various immune cells from *Slc29a3*^−/−^ mice (related to Figs. 1 and 3). Fig. S4 shows that *Slc29a3*^−/−^ mice do not produce inflammatory cytokines and type-I IFNs (related to Fig. 3). Fig. S4 also shows the cytokine production by *Slc29a3*^−/−^ macrophages in response to polyU (related to Fig. 4). Fig. S5 shows the expression levels of ITAM adaptors (related to Fig. 5), and the data from a patient carrying the S184R *SLC29A3* mutation and from healthy subjects (related to Fig. 6). Also shown is the strategy for generating human TLR7 and TLR8 Tg mice, along with the data from *Slc29a3*^‒/‒^*Tlr7*^‒/‒^ human TLR7 Tg mice (related to Fig. 6).

## Data Availability

All the RNA sequence data analyzed in this manuscript were deposited in the NCBI Gene Expression Omnibus database under accession number GSE234367. All other data are available in the main text or the supplementary material.
